# Effects of an app-augmented cognitive behavioral therapy using the Journaling App for Youth (JAY) for adolescents with internalizing disorders—a study protocol

**DOI:** 10.1186/s13063-026-09806-0

**Published:** 2026-06-11

**Authors:** Anja Görtz-Dorten, Lisa Schindler, Johanna Klee, Laura Wähnke, Manfred Döpfner

**Affiliations:** 1 Department of Child and Adolescent Psychiatry, Psychosomatics and Psychotherapy, Faculty of Medicine and University Hospital Cologne, University of Cologne, Robert-Koch-Straße 10, Cologne, 50931 Germany; 2 Center for Child and Adolescent Cognitive Behavior Therapy (CEKIP), Faculty of Medicine and University Hospital Cologne, University of Cologne, Pohligstraße 9, 50969 Cologne, Germany

**Keywords:** Smartphone app, Adolescents, Anxiety, Depression, Cognitive-behavioural therapy, Therapy homework adherence

## Abstract

**Background:**

Internalizing disorders such as anxiety and depression can be especially severe during adolescence, presenting significant challenges for affected individuals. While cognitive-behavioural therapy (CBT) has shown promise as an effective treatment for these conditions, a significant proportion of youth do not benefit sufficiently from CBT. One critical factor that may influence the effectiveness of CBT is adherence to therapy homework, with evidence suggesting a relationship between homework completion and therapy outcomes, including symptom reduction. In this context, smartphone apps have emerged as a potentially helpful tool to enhance adherence by providing engaging ways for adolescents to complete their therapy homework. However, in Germany, there is currently a lack of evaluated smartphone apps that can be deployed as adjuncts to CBT for adolescents.

**Method:**

This study is designed as a randomized controlled trial (RCT). It will evaluate the effects of the JAY smartphone app on therapy homework adherence during CBT for adolescents with internalizing disorders (*n* = 35) compared to CBT with standard paper-and-pencil homework (*n* = 35).

**Discussion:**

The findings of the JAY trial may contribute to the understanding of how smartphone apps can be integrated into CBT for adolescents to enhance therapy homework adherence and ultimately treatment outcomes.

**Trial registration:**

German Clinical Trials Register (DRKS) DRKS00026623. Registered on 13th October 2023, https://drks.de/search/de/trial/DRKS00026623/details.

**Supplementary Information:**

The online version contains supplementary material available at 10.1186/s13063-026-09806-0.

## Background

Anxiety and depressive disorders are highly prevalent during adolescence [1, 2], and the rates of these disorders have significantly increased as a consequence of the COVID-19 pandemic [3, 4]. They frequently co-occur and show a significant convergence of symptoms [5, 6]. Adolescents with anxiety or depressive disorders show lower social and academic functioning than unaffected peers [7, 8], and even more concerningly, have a higher risk of suicidality [9–11], which is a leading cause of death in young people [12]. The presence of affective disorders in adolescence and young adulthood further predicts poor health outcomes and functioning in adulthood [13, 14]. Considering the high prevalence and adverse impacts of these disorders, effective treatment for affected adolescents is crucial.

According to scientific consensus, cognitive-behavioural therapy (CBT) is the method of choice for treating anxiety and depressive disorders in both adults and youth (e.g., [15–17]). Nevertheless, a careful examination of the treatment effects of psychotherapy for depressed and anxious youth reveals only modest effect sizes. A significant proportion of affected youth are non-responders to therapy or continue to fulfill the criteria for a psychiatric diagnosis at the end of therapy [18–21]. Furthermore, findings regarding the long-term effects of CBT are mixed [15, 22].

The main influence of CBT lies in inducing cognitive and behavioural changes. This can be accomplished through cognitive restructuring as well as exposure and behavioural interventions [23]. One important component of CBT is therapeutic homework, which is a collective term to describe any activity that was developed within the session and should be completed by the patient between sessions. In the treatment of anxiety and depressive disorders, homework may include, in particular, journaling, exposure exercises in a natural setting, and activity scheduling [24]. Homework helps to generalize and maintain the skills acquired during therapy sessions [25, 26]. Moreover, patients are able to observe and document problematic situations throughout the week and bring them to the therapy session to discuss them with the therapist. Research in adults has shown that the amount of homework that patients complete and the quality of therapy homework adherence (THA) significantly predict treatment outcomes in terms of the reduction of anxiety and depressive symptoms (e.g., [27–29]). There is also evidence that this applies to adolescent patients with depression [30] but not for the treatment of adolescent anxiety [31].

In summary, THA is an important factor for treatment outcomes, with low THA potentially contributing to insufficient effectiveness of CBT. Indeed, studies have shown that low THA is a common phenomenon in cognitive therapy, especially in adolescents [32, 33]. For example, a study measuring homework compliance in adolescents with depression receiving CBT reported a homework completion rate of only 56%, and a decline during the therapy process was noted [34]. Non-adherence is influenced by patient, therapist, and task factors, but an empirical evaluation is impeded by various methodological difficulties [32, 35]. Nevertheless, some experience-based recommendations to improve THA can be made. For instance, homework should be meaningful, relevant to the central goals of therapy, salient to the focus of the session, agreeable to the therapist and patient, appropriate to the patient’s sociocultural context, doable, small, have a clear rationale and back-up plan, and should be practiced in session [36, 37]. It is also recommended that a time and place to complete the homework should be defined in advance [36, 37]. Accordingly, a qualitative study found that adolescent patients often perceive homework as too difficult or time-consuming and that they lack the motivation to complete it [38].

Smartphone apps may provide a way of improving mental health treatment in general and THA specifically [39–42]. Modern-day youth are growing up in an increasingly digital environment, gaining internet access at progressively younger ages [43]. Notably, around 96% of German adolescents aged 12–19 years have their own smartphone [44]. As such, mobile technology might represent a promising platform for enhancing mental health treatment [45].

In particular, smartphone apps can make psychoeducational content more accessible and facilitate in-the-moment self-assessments (such as documentation of thoughts and feelings), thus reducing recall bias. Moreover, they can support the practice of coping, relaxation, problem-solving skills, and exposure assignments. Mobile technology has certain advantages over paper-and-pencil-based homework. For instance, apps can respond adaptively to the patient’s input and give personalized feedback. They can send scheduled reminders in the form of push notifications and can include a display of progress as well as reward systems [46, 47]. The feature of in-the-moment assessments in natural settings, also known as ecological momentary assessment (EMA), might also enhance research on the feelings, thoughts, and behaviour of children and adolescents [48].

New apps are consistently being developed and introduced, and need to be scientifically evaluated in order to be used safely. Therefore, several (systematic and meta-) reviews have attempted to depict the current scientific basis of the employment of apps in the field of mental health. These reviews have analyzed apps used as an adjunct to psychotherapy [49] and stand-alone apps that are not integrated into face-to-face psychotherapy [50, 51]. Further research has focused on apps for youth and apps used in patients with anxiety or depression [52–55].

The functions of the apps analyzed in the reviews include monitoring symptoms (including mood, thoughts, and behaviour) as well as providing interventions, such as psychoeducation, exposure, response prevention, skills practice (e.g., mindfulness exercises), and promoting positive behaviours [53, 56, 57]. Monitoring functions are predominantly reported, with some apps even being limited to these functions [50, 54, 56]. The reviews show that most web or mobile apps are designed as stand-alone apps rather than serving as an adjunct to psychotherapy, despite the limitations of stand-alone apps [49, 50, 55]. Overall, the evidence on app usage seems to cautiously point in a positive direction, as most studies conclude that apps are safe, effective, easy to use, and well-received by patients and therapists in terms of acceptability and satisfaction [49, 55]. However, a decline in patient engagement over time has been noted, especially for assessment functions, albeit less so for adjunctive apps [49, 52, 55]. Adjunctive app use is also assumed to yield better longer-term outcomes [49, 50]. Monitoring activities seem to be undertaken more consistently via apps than on paper [56].

Although many apps claim to be based on CBT, the term is often used loosely and does not necessarily imply that an app draws on an evidence-based framework. Various authors have underlined that apps should be developed by trained psychologists and scientifically evaluated [49, 58].

A further important aspect to be considered is data privacy. Apps should not make patients’ data available to third parties, which would threaten confidentiality, and the transfer of data to the therapist should only take place with the patient’s informed consent [50, 56].

Overall, the reviews conclude that the evidence base for mobile apps is rather thin [52], especially regarding their use in child and adolescent psychotherapy [56]. Some apps lack any scientific evaluation, and high-quality studies such as randomized controlled trials (RCT) with active control groups are scarce [49, 52, 55], particularly in the case of adjunctive apps [49]. Furthermore, a more systematic investigation is needed to understand how smartphone technology provides therapeutic benefits. This includes examining common features of therapy apps, such as behaviour diaries, push notifications, and clinical information [59].

Research on app usage between therapy sessions and the effects of app usage on THA is even more scarce, even though it is recognized as having great potential [40, 46, 60]. Apps designed for adults that target THA include CBT MobileWork© for depression [61], CBT Assistant for social anxiety disorder [62], and PE Coach for post-traumatic stress disorder [63–65]. However, RCTs to prove the purported effects of these apps have yet to be conducted.

To the best of our knowledge, only one study to date has specifically explored the use of app-augmented CBT for the treatment of anxiety and depressive disorders in young individuals targeting THA [66]. In an ongoing multisite, randomized controlled pragmatic clinical trial, the authors are investigating the impact of a smartphone app on homework completion. They hypothesize that participants using the app-enhanced treatment will show higher homework compliance. Given the currently low compliance rates, the app is anticipated to have moderate effects. Since no results from this project were available at the time of our study protocol submission, we can only discuss aspects of the study design, such as sample size, without insights into the outcomes or effectiveness of the design.

Therefore, the purpose of this study is to address this research gap by evaluating a smartphone-enhanced CBT intervention designed for youth with various disorders, specifically focusing on anxiety and depressive disorders in this study, using a newly developed app (Journaling App for Youth, JAY) [67]. The primary aim of the JAY study is to investigate whether individuals who undergo a therapeutic intervention enhanced by smartphone usage show notably greater compliance in terms of both the (i) quantity and (ii) quality of completed therapy homework assignments compared to individuals who undergo a similar treatment without smartphone app support.

The following hypotheses will be investigated:

1. The experimental group (CBT + JAY) will show a significantly higher overall THA score compared to the active control group (CBT) throughout the intervention phases, as assessed by quantitative aspects of therapy homework (relative ratio of completed and required entries) as well as qualitative aspects which together as a composite score constitute the primary outcome. The qualitative aspects (content fit, thoroughness, competence of behaviour/thoughts, and clinical relevance) will be evaluated by multiple independent raters using a rating system that defines the assessment criteria.
2. The experimental group (CBT + JAY) will show higher THA compared to the active control group (CBT) as assessed using the clinician-rated Questionnaire for Therapy Adherence after each therapy session [68].
3. The experimental group (CBT + JAY) will show a greater reduction in internalizing symptoms compared to the active control group (CBT) according to self-rating, as measured using the Internalizing Problems scale of the Youth Self-Report 11–18 (YSR/11-18R) [69, 70] as well as the self-report symptom checklists for anxiety (SCL-AD-S) and depression (SCL-DEP-S) from the “Diagnostic system for mental disorders based on ICD-10/DSM-5 for children and adolescents” (DISYPS-III) [71] and using an individual problem list.
4. The experimental group (CBT + JAY) will show a greater reduction in internalizing symptoms compared to the active control group (CBT) according to caregiver rating, as measured using the Internalizing Problems scale of the caregiver-report Child Behaviour Checklist (CBCL/6-18R) [69, 70] as well as the caregiver-report scales for anxiety (SCL-AD-P) and depression (SCL-DEP-P) from the DISYPS-III [71].
5. The experimental group (CBT + JAY) will show a greater reduction in internalizing symptoms compared to the active-control group (CBT) according to clinical rating, as assessed by a clinician using a structured clinical interview (ILF-INTERNAL) from the DISYPS-III [72].
6. The experimental group (CBT + JAY) will show a stronger reduction in comorbid symptoms as measured using the scales Externalizing Problems and Total Problems of the self-report YSR/11-18R and the caregiver-report CBCL/6-18R [69, 70].
7. Patients in the experimental group (CBT + JAY) will show a greater improvement in quality of life, sense of global self-worth, and emotion regulation strategies according to self-report compared to patients in the control group (CBT), as assessed by the Revised Questionnaire for Health-Related Quality of Life Assessment in Children (KINDL^R^) [73], by a modified German version of the Self-Perception Profile for Children called the Harter scale [74, 75], and by a modified version of the Questionnaire to Assess Children’s and Adolescents’ Emotion Regulation Strategies (FEEL-KJ) [76], respectively.
8. Patients in the experimental group (CBT + JAY) will show greater treatment satisfaction than those in the control group (CBT), as measured by a modified version of the self-report Client Satisfaction Questionnaire (ZUF-8-M patient) [77, 78].

A comprehensive description of the clinical assessment tools mentioned above can be found in the “ Secondary outcomes” section. Additionally, the following research questions will be addressed:

1. How feasible is the JAY smartphone app, and how satisfied are patients regarding its use?
2. What are the clinical implications of symptom changes observed in the experimental group relative to those in the control group?
3. Which moderators and predictors (such as gender and age) can be identified to predict treatment outcomes?

## Methods

### Trial design

The Journaling App for Youth (JAY) study is designed as an interventional randomized controlled trial (RCT). It will include two parallel treatment arms: (1) cognitive-behavioural therapy (CBT) employing the Journaling App for Youth (CBT + JAY) or (2) cognitive-behavioural therapy employing traditional paper-and-pencil methods (CBT).

**Fig. 1:**
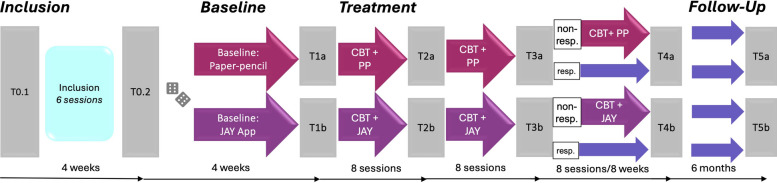
Study design of the JAY trial

Figure 1 presents the trial design for the JAY study. Patients and their caregivers will be contacted by telephone for a preliminary screening, followed by an in-person screening and information session. Upon agreeing to participate, patients and caregivers will complete the first online questionnaires (T0). Subsequently, diagnostic sessions and sessions to introduce the patient to their therapist will be conducted.

Following these sessions, patients will be randomly allocated to one of the two treatment arms. After randomization, a baseline phase will take place, followed by the T1 assessment. In the subsequent treatment phase, patients in both groups will receive state-of-the-art CBT. In the treatment group (CBT + JAY), homework and assessments will be conducted through the JAY app, while in the active control group (CBT), equivalent tasks will be completed on paper. Each treatment phase will consist of eight sessions with the patient and up to four sessions with a caregiver. The first treatment phase will be followed by the T2 assessment. The final main assessment (T3) will take place after the second treatment phase. Depending on the clinical significance of remaining internalizing symptoms, patients will then either continue with a third treatment phase, which will be followed by the T4 assessment, or the intervention will be stopped and they will proceed to a first follow-up assessment after 8 weeks (T4). A subsequent follow-up evaluation (T5) for all patients will be scheduled 6 months after T4.

## Participants, interventions, and outcomes

### Study setting

The JAY trial will be a single-center study located at the Center for Child and Adolescent Cognitive Behavior Therapy (CEKIP), Faculty of Medicine and University Hospital Cologne, University of Cologne.

### Eligibility criteria

Eligible participants will meet the following criteria: (1) adolescents aged between 13;0 and 17;11 years at T1; (2) diagnosed with one of the specified clinical conditions according to the 10th International Statistical Classification of Diseases and Related Health Problems (ICD-10) [79], including depressive episode (F32.0, F32.1, F32.8, F32.9), anxiety disorders (F40.00, F40.01, F40.1, F40.8, F41.0, F41.1, F41.2), emotional disorder with onset specific to childhood (F93.0, F93.2), and adjustment disorder (F43.2), as assessed by a clinician using a structured clinical interview (DISYPS-ILF INTERNAL) [72]; (3) self-reported internalizing symptoms with a clinically relevant score (*T* value ≥ 60) on the Internalizing scale of the YSR/11-18R [69]; and (4) willingness and ability to participate in the intervention, including sufficient German-language skills and smartphone competences demonstrated by the patient.

Patients will be excluded if any of the following exclusion criteria are met: (1) presence of autism spectrum disorder (F84) or another severe comorbid disorder identified as clinically predominant; (2) adolescent’s intelligence level below average (intelligence quotient (IQ) < 80), determined using a standard intelligence test (German version of the Culture Fair Intelligence Test (CFT 20-R) [80]); (3) critical escalations of symptoms indicative of the need for inpatient treatment; (4) concurrent participation in additional outpatient treatment; (5) indication for initiating new pharmacotherapy or adjusting the dosage of existing pharmacotherapy at the time of the intervention.

### Consent

Informed consent will be obtained from patients and caregivers in written and verbal form prior to screening. For detailed information, please refer to Additional file 2: Model of written informed consent. Participants will have the opportunity to address any questions regarding the study with research staff members. Participation in the study requires assent and informed consent from both the patient and their caregivers. Data entry and processing will take place upon receipt of signed informed consent.

### Interventions

#### Explanation for the choice of comparators

The choice of an active control group rather than a placebo or no-treatment control group ensures that both groups will receive a recognised and effective form of treatment. This design will allow us to assess the added value of the app, if any, beyond the established benefits of CBT and therapy homework, thereby providing a meaningful comparison that can inform future clinical practice.

#### Intervention description

In the randomized control trial, each participant will receive cognitive behavioral therapy conforming to German clinical guidelines and based on international evidence-based treatment manuals (e.g., the SELBST program [81], THAZ—Social Anxiety [82], CBT for Depression in Childhood and Adolescence [83]). Participants will undergo 16 to 24 weekly therapy sessions, and up to 12 optional caregiver sessions will be provided (a maximum of four per treatment phase).

A personalized treatment plan will be created for each patient. Common elements of treatment include establishing rapport and motivation, resource activation, developing a mutual understanding of the disorder and its causes, psychoeducation, situational analysis, and setting treatment goals.

For the treatment of depressive symptoms, tailored interventions may include resource activation for low self-esteem, promoting activities for patients lacking positive experiences, problem-solving training for inadequate conflict resolution, cognitive restructuring for dysfunctional thought patterns, third-wave techniques for managing ruminative thoughts, and self-reinforcement for insufficient self-care.

Anxiety treatment may involve cognitive restructuring for dysfunctional thoughts, graduated exposure therapy combined with positive reinforcement to address avoidance behaviours, and competence training for lacking skills (such as social skills).

At the conclusion of each treatment, there will be an evaluation, relapse prevention strategies, and emotional detachment from the therapist.

The study therapies are conducted by child and adolescent therapists in training. Therapists need to be familiar with the app’s features and comfortable in incorporating the app into the therapy sessions. Otherwise, implementation of and fidelity to the intervention might be threatened. To limit this risk, therapists will receive a comprehensive introductory session to the app as well as ongoing support on how to integrate it into their therapy sessions effectively. The study staff will deliver this introductory session and offer continuous technical assistance. The supervisor will offer guidance to therapists on the effective integration of the app into therapy sessions. Additionally, a comprehensive user manual will be provided [67].

#### JAY smartphone app

**Fig. 2:**
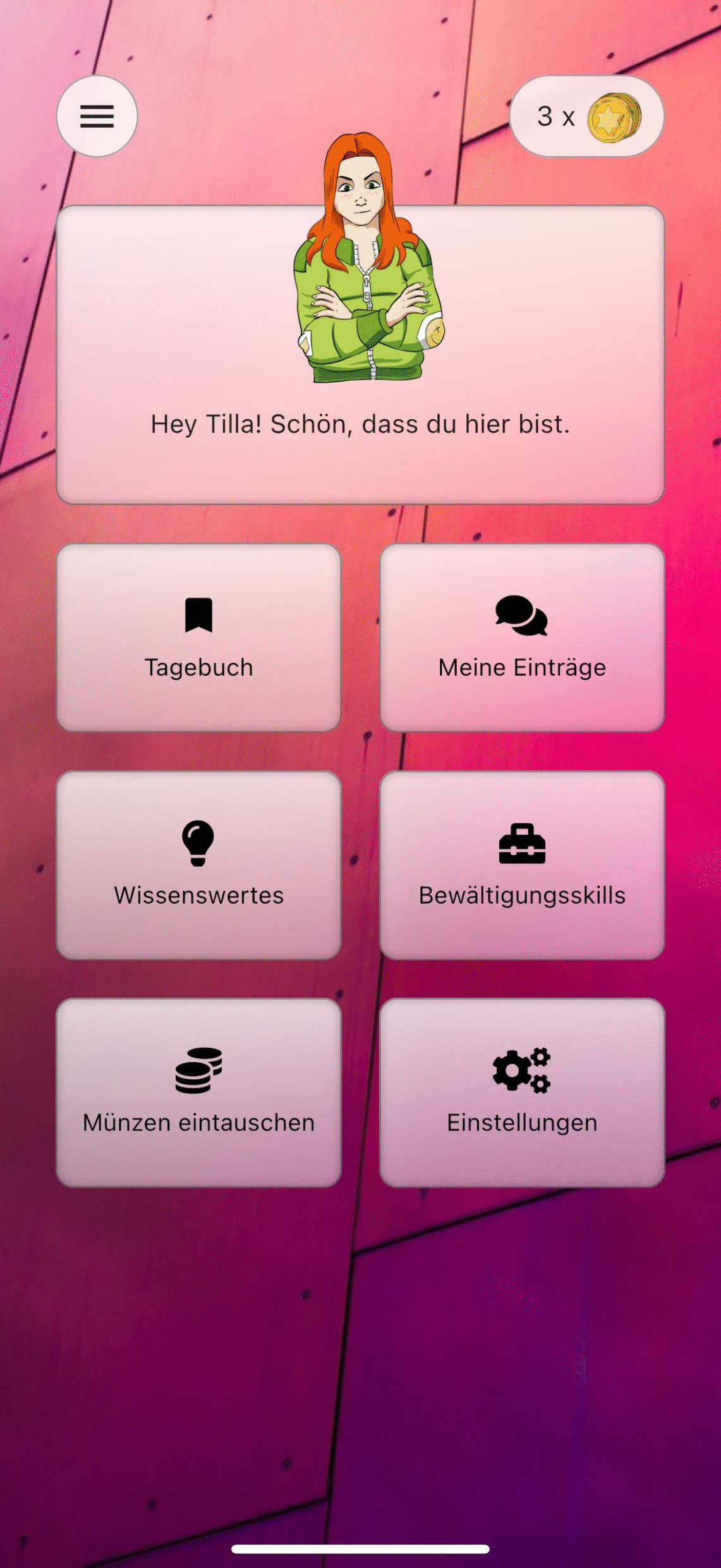
Main menu of the JAY app (version 1.1.0)

During the CBT treatment, adolescents in the experimental group will use the JAY app [67] (see Fig. 2), which is designed for adolescents aged between 13 and 17 years and can be used in the treatment of mental disorders, e.g., anxiety and depressive disorders, conduct disorder, attention-deficit/hyperactivity disorder, and obsessive-compulsive disorder. The current study focuses on patients diagnosed with at least one of the two internalizing disorders anxiety and depressive disorders. The JAY app can be combined with a variety of CBT manuals and is designed to facilitate the implementation of therapeutic homework and the transfer of coping strategies into patients’ daily lives. It can also be used for diagnostic and progress monitoring purposes. The app can be customized with regard to design (according to the patient’s wishes), difficulty of tasks (depending on therapy progress), and individual questions defined by the therapist. The different functions of the app are described in greater detail below [67].

The *design function* allows patients to individualize the app’s interface, including colours, backgrounds, and an avatar that interacts with the patient. This is supposed to increase therapy motivation.

The *psychoeducation function* supports youth-friendly psychoeducation involving explanatory graphics. These illustrate the causes of problem behaviour (“This is how it may be”), introduce CBT interventions, and allow an outlook for improvement (“This is how it may become”).

**Fig. 3:**
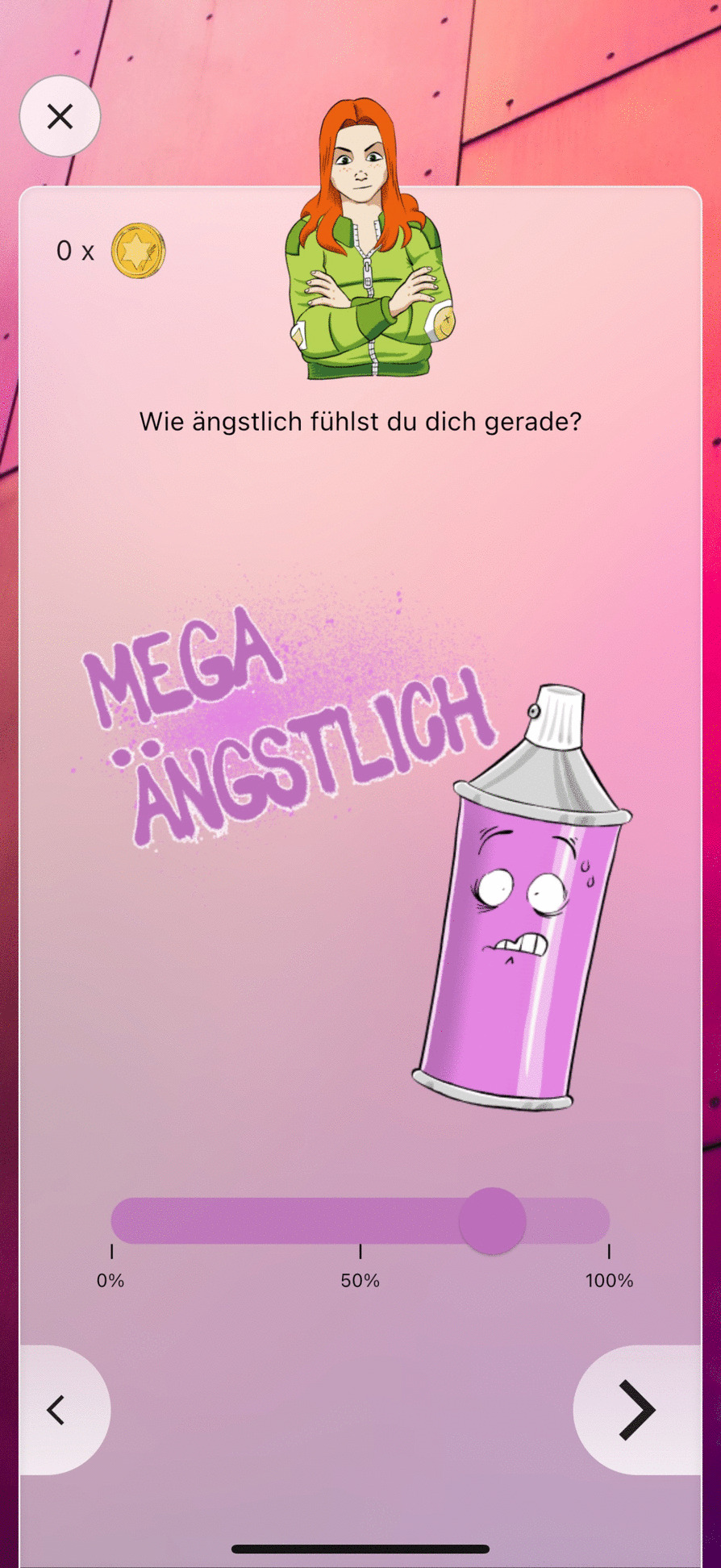
Momentary assessment function of the JAY app (version 1.1.0)

The *momentary assessment function* (see Fig. 3) assesses the intensity of 18 affective and mental states (e.g., happy, afraid, relaxed, sad), 16 of which will be used in the current study. This is a tool for self-observation of mood in real time, which can yield data without retrospective biases. The collected data can be valuable for clinicians in terms of diagnosing mood disorders or tracking the effectiveness of treatment over time. Moreover, the function can make individual queries, allowing for specific questions tailored to the patient’s situation. Queries will only occur at predetermined times upon which the patient and therapist have agreed, and cannot otherwise be triggered. This ensures that mood data are collected consistently and at relevant intervals. Once alerted, the user has a time window of 1 h to complete the mood assessment, thus providing flexibility while still capturing data within a relevant time frame.

**Fig. 4:**
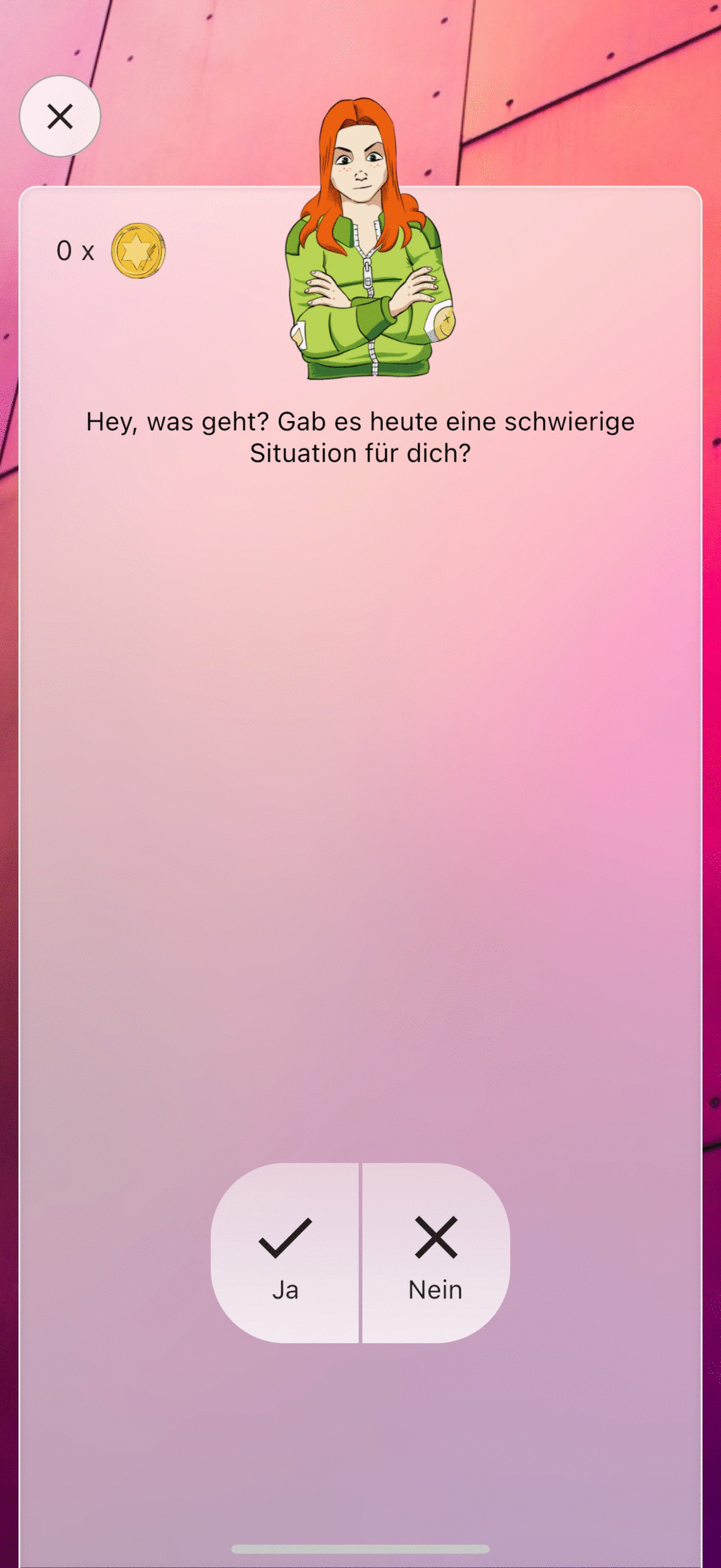
Diary function of the JAY app (version 1.1.0)

The *diary function* (see Fig. 4) can be used to report on difficult situations that patients encounter in their everyday lives, including cognition, behaviour, and consequences of behaviour, among other things. Diary entries can be made during or after the situation, allowing for a better transfer to therapy. The app specifically prompts the patient to analyze the situation, guiding the process. This function is supposed to support the training of the ability to reflect. First, questions about difficult situations are asked. If the patient indicates that a difficult situation has not occurred, a positive experience can be reported instead. When activated, the diary function is accessible to patients at any time in the home menu. The content and structure of the prompts can be modified in terms of disorder-specific questions and with respect to the complexity of the questions. For each question that requires input, the patient can choose between a text entry and a video recording.

The *reminder function* prompts the patient to start his/her therapy homework that has been individually agreed upon. A first notification is set up as reminder to accept the challenge, and a second notification prompts the patient to document whether the challenge was accepted, how it went, or what caused it to fail.

The *problem-solving function* aims to help patients develop problem-solving skills by acting as their own “director” in challenging situations. A problematic situation can be defined in the app. The patient is then confronted with the situation and asked for a strategy to solve the problem. A specific action goal can be set, e.g., to calm oneself down.

The *coping skills function* provides patients with a structured and accessible way to learn and apply various coping strategies for managing stress, emotions, and challenging situations. Those coping strategies are presented within four overarching categories: (1) thought-related skills, (2) relaxation, action, and sensory-related skills, (3) problem-solving skills, and (4) reward and helping skills. Various subcategories are available, as well as the option to record the use of skills in a skills diary. Once activated in the settings, this function can be accessed autonomously by the patients. Individual skills can be added in order to personalize the experience.

The *reinforcement function* serves as a motivational system that rewards adolescents for actively engaging with the app’s features. By engaging with the various functions, patients can earn coins. These can be redeemed in the app’s virtual store for an app-internal game (Blocks or Snake) or for three individually determined wishes with different values.

#### Modifications

The study participation of single subjects will be terminated early, and subjects will be defined as dropouts, if at least one of the following conditions is met: (i) at least one parent/caregiver withdraws the informed consent, (ii) the patient withdraws the informed consent, (iii) the patient requires inpatient treatment or another kind of outpatient treatment in order to safeguard his or her well-being, which may be affected by continued trial participation.

The principal investigators may decide to terminate the entire trial if less than 50% of the planned sample size can be recruited despite additional recruitment strategies.

#### Adherence

Patients’ adherence to the study protocol will be ensured through the following measures: (i) During the patients’ weekly therapy sessions, adherence to the study protocol will be discussed alongside their regular progress reviews, including homework completion. Since the study data (e.g., homework completion rates, symptom tracking) are directly informative for the therapist’s clinical planning, this discussion naturally aligns with the therapeutic goals. (ii) Therapists will document adherence-related discussions and any protocol deviations in the Adherence Questionnaire, which will be regularly reviewed by the research team.

Treatment fidelity on the part of the therapists will be ensured through several measures: (i) Therapists will complete a structured protocol after each session, and (ii) a supervision session with the PI will be scheduled for every fourth therapy session.

#### Concomitant care

During the trial, concurrent psychotherapy will be prohibited for participants. This is because receiving additional psychotherapy would not only conflict with the study’s treatment protocol but is also contrary to clinical recommendations. If a participant begins concurrent psychotherapy during the trial, this will result in their dropout from the study in order to maintain the integrity of the trial’s outcomes and ensure consistency in the treatment being evaluated.

### Outcomes

#### Primary outcome

The primary outcome of the JAY trial will be patients’ THA. THA will consist of an overall THA score including the quantity and quality of therapy homework. Patients in both treatment arms will be asked to complete homework assignments between the therapy sessions. This will include at least two diary entries and one individually assigned challenge that has to be documented (via the app or on paper, depending on the study group). Patients will complete at least 16 sessions of therapy, and THA can be assessed after 15 sessions. Non-responders will complete a further eight sessions, so that THA can be rated 23 times over 23 weeks.

The quantity of therapy homework will be assessed according to the relative ratio of completed to requested entries that are implemented in the JAY app or the paper-and-pencil sheets between the treatment sessions. The relative ratio will be computed by dividing the number of completed entries by the number of requested entries and will thus range from 0 to 1. The quality of the entries will be assessed by a pool of clinicians based on the app entries or the homework sheets according to a rating system. Criteria of the rating system include (1) content fit, (2) thoroughness, (3) competence of behaviour/thoughts, and (4) clinical relevance. As these quality data might be subjectively biased, they will be further evaluated by five independent clinical raters on a 4-point Likert scale (0 = poor quality, 1 = low quality, 2 = largely good quality, 3 = good quality). The clinical ratings will be conducted based on a rating manual specifying the quality criteria. These aspects do not necessitate any further subjective interpretation by the evaluating clinician, thereby minimizing the potential for bias. Intraclass correlation coefficients (ICC) will be computed to assess the interrater agreement, applying a two-way mixed model for consistency and absolute agreement. In a previous study on the AUTHARK app—the child version of the JAY app—a preliminary analysis of 15 app and paper-and-pencil entries was conducted and interrater reliability for five independent raters was assessed [41]. Consistency for this method was evaluated as “very good” (0.95). The values for absolute agreement ranged between 0.73 (“average”) and 0.87 (“good”) [84]. To calculate the overall THA score, the product of the relative ratio (e.g., 17 completed entries with 30 required entries (17/30 = 0.57) and the average quality score (i.e., from 0 to 3) will be computed. Therefore, the THA score ranges between 0 and 3.

Initially, a descriptive report will be provided for each assessment time point, detailing the quantity of finalized app entries or self-observation sheets, along with the outcomes of the coding process. Subsequently, we will display the change of mean values for each specific aspect over time, both within and across the different groups.

#### Secondary outcomes

Secondary outcomes include:

1. The quantity of therapy homework, as assessed by the relative ratio of completed to requested entries within the JAY app or on the paper-and-pencil sheets between the treatment sessions.
2. Quality of the entries, as rated by a pool of clinicians based on the app entries or the homework sheets according to the rating system mentioned in the previous section.
3. Patient-rated symptoms of anxiety and depression, assessed by the Internalizing Problems scale of the self-report YSR/11-18R [69, 70] as well as the self-report scales for anxiety (SCL-AD-S) and for depression (SCL-DEP-S) from the DISYPS-III [71]. Reliability and validity as well as sensitivity to change of the German version of the YSR/11-18R and the DISYPS-III scales have already been demonstrated in German samples [70, 71]. The Internalizing Problems scale of the YSR/11-18R will be assessed at every measurement time point and the SCL-AD-S and SCL-DEP-S will be assessed at T0 and T3.
4. Caregiver-rated symptoms of anxiety and depression, assessed by the Internalizing Problems scale of the caregiver-report CBCL/6-18R [69, 70] as well as the caregiver-report scales for anxiety (SCL-AD-P) and for depression (SCL-DEP-P) from the DISYPS-III [71]. Reliability and validity as well as sensitivity to change of the German version of the CBCL and the DISYPS-III scales have already been demonstrated in German samples [70, 71]. The Internalizing Problems scale of the CBCL/6-18R will be assessed at every measurement time point, and the SCL-AD-P and SCL-DEP-P will be assessed at T0 and T3.
5. Clinician-rated symptoms of anxiety and depression based on the ILF-INTERNAL from the DISYPS [72], assessed at T0 and T3.
6. Individually defined problem behaviours were rated by the patients using an individual problem list (IPL) in every therapy session. The problem list allows for the assessment of four targeted issues, rated on a 5-point Likert scale, and of the burden of the problems, rated on a 10-point Likert scale. Individual problem lists have been shown to be reliable, valid, and sensitive to change [85].
7. Patient- and caregiver-rated comorbid symptoms and overall behavioural and emotional problems, measured using the remaining scales Externalizing Problems and Total Problems of the self-report YSR/11-18R and the caregiver-report CBCL/6-18R [69, 70]. Reliability and validity as well as sensitivity to change of the German version of these scales have been demonstrated in German samples [70]. These outcomes will be measured at T0 and T3.
8. Patients’ adherence during the weekly therapy sessions (e.g., engagement in tasks and activities) will be measured using the clinician-rated Adherence Questionnaire [68], which will be assessed after every therapy session. This was adapted from the AUTHARK trial [41], in which good internal consistencies were reported.
9. Quality of life, assessed using the Revised Questionnaire for Health-Related Quality of Life Assessment in Children and Adolescents (KINDL^R^) [73], which evaluates aspects related to physical and psychological well-being, the adolescent’s self-worth, as well as friendships and family relationships. Reliability and validity as well as sensitivity to change of scale have been demonstrated in German samples [86, 87]. This outcome will be assessed at T0 and T3.
10.  Sense of global self-worth, assessed using a modified German version of the Self-Perception Profile for Children, called the Harter scale [74, 75], which evaluates perceptions of cognitive and physical competence, peer acceptance, physical appearance, and self-worth. Reliability and validity of the original scale were demonstrated in a German clinical sample [88]. This outcome will be assessed at T0 and T3.
11.  Emotion regulation strategies were assessed using a modified version of the Questionnaire to Assess Children’s and Adolescents’ Emotion Regulation Strategies (FEEL-KJ) [76], which evaluates adaptive and maladaptive strategies to regulate feelings of anxiety and sadness. Reliability and validity have been demonstrated [76]. This outcome will be assessed at T0 and T3.
12.  Retrospective mood assessments will be conducted in every week in which the patient has used the momentary assessment of the JAY app or the paper-and-pencil equivalent. All 16 momentary affective and mental states assessed during the week will be rated retrospectively for the same week on a scale from 0 to 100.
13.  Patient-rated satisfaction with the treatment, assessed using a modified version of the self-report Client Satisfaction Questionnaire (ZUF-8-M patient) [77, 78], which has been shown to be reliable, valid, and sensitive to change [89]. Satisfaction will be assessed once, at T3.
14.  Patient-rated usability of the JAY app, assessed using a German translation of the System Usability Scale (SUS), which has been shown to be reliable, usable, and valid [90]. The scale measures effectiveness, efficiency, and satisfaction of and with a system. Usability will be assessed for patients in the experimental group once, at T3.
15.  Life events in the domains of school, family, and friendships as well as illness, accidents, and losses, as measured by the Zurich Life-Event List (ZLEL), which has been shown to be reliable and valid [91]. This measure will be applied once, at the follow-up (T5) in this study.

### Participant timeline

The participant timeline and measurement schedule for the JAY trial will include four key stages: a screening process, a baseline phase, an intervention period, and a follow-up assessment. The screening process will assess whether families meet the study’s inclusion and exclusion criteria in several steps. First, families will be contacted by telephone for a brief standardized screening. If the initial criteria are met, the patient and a caregiver will be invited for an in-depth screening and information session. Participants who agree to join the study will complete initial questionnaires and undergo three diagnostic sessions to thoroughly assess eligibility (T0). This will be followed by randomization to the experimental or control group. After this, three sessions will be conducted to introduce the patient to their therapist, focusing on building a strong patient-therapist relationship and providing psychoeducation.

The screening process will be followed by a baseline phase that does not include any sessions with the study staff or therapist. Participants will use the JAY app [67] or paper-and-pencil sheets for the purpose of self-observation.

The pre-treatment assessment (T1) will mark the end of the baseline and beginning of the treatment phase. The first part of the treatment phase will involve eight sessions of cognitive-behavioural therapy and up to four sessions with caregivers, followed by an intermediate assessment (T2). The second part of the treatment phase will likewise consist of eight patient sessions and up to four caregiver sessions. Subsequently, another main assessment (T3) will follow. Non-responders to the treatment will be identified using the Internalizing scale from the YSR/11-18R [69] and will continue to receive CBT in a third part of the treatment phase. This will then be followed by a final assessment (T4). Responders to the treatment, as indicated by a lack of significant internalizing symptoms at the main assessment (T3), will conclude their participation in the study. Eight weeks after the final therapy session, they will engage in an initial follow-up assessment (T4).

Finally, every family will complete a follow-up survey (T5) 6 months after T4.

The patient will visit the study site four times for information, screening, and diagnostics, twice for a clinical interview at T3, and 19 (responders) or 27 (non-responders) times for sessions with the therapist. Caregivers will visit the study site for the screening/information session, once for taking the patient’s history, and up to eight times (responders) or 12 times (non-responders) for sessions with the therapist.

**Fig. 5:**
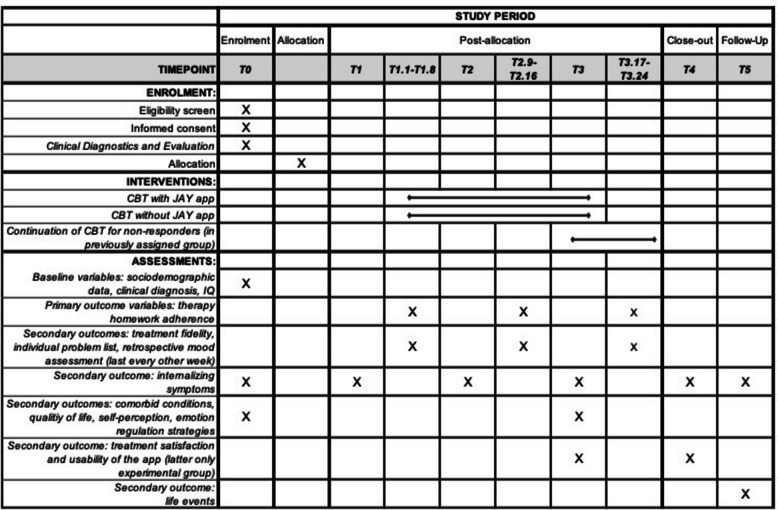
SPIRIT figure

Therapy homework adherence, treatment fidelity, and an individual problem list will be assessed every session and a retrospective mood assessment will be conducted every other week. At the main assessment points (T0, T3), internalizing symptoms will be assessed using self- and caregiver-report questionnaires as well as clinical interviews. Comorbid conditions will be assessed only using self- and caregiver-report questionnaires. At the intermediate assessment (T2), internalizing symptoms will be assessed by the patient and the caregiver (see Fig. 5).

### Sample size

This study will include *N* = 70 adolescents (35/35) in order to explore the impact of smartphone-augmented CBT on therapy homework adherence among adolescents with depressive and anxiety disorders. Based on a recent systematic review [49], which analyzed the effects of adjunctive apps on therapy across 19 studies, the sample size of 70 in our study lies within the range of reported sample sizes, thus providing a solid basis for comparing the efficacy of adjunctive mobile applications in therapeutic settings. Additionally, a study employing a comparable methodology, which involves a comparison of two groups of 35 participants over four evaluation periods (including before treatment, after treatment, and follow-up assessments), anticipates moderate impacts of their app specifically on therapy homework adherence. For the comparison between groups across four measurement time points, a power analysis indicated a power of 0.84 with a sample size of 35 per group (app vs. no app) at an α level of 0.5 [66]. Additionally, the statistical power of a sample can be enhanced through a large number of observations per participant [34, 92]. In our research, THA will be evaluated for a minimum of three homework tasks per week over approximately 16 weeks.

### Recruitment

Patients for the JAY trial will be recruited from (1) the outpatient units of the Department of Child and Adolescent Psychiatry and of CEKIP at the University Hospital Cologne, and (2) paediatric and psychiatric practices in Cologne, Germany.

## Allocation

### Sequence generation

The study will involve randomly allocating participants to different treatment groups. In the experimental group, 35 patients will receive traditional CBT supplemented by the JAY app [67]; in the control group, 35 patients will receive CBT without the app, using traditional paper-and-pencil homework. Participants will be randomized using computerized block randomization with a block size of four.

### Concealment mechanism

Allocation concealment will be ensured, as randomization will only take place after consent has been obtained from both caregivers and patients and it is confirmed that all inclusion and exclusion criteria are fully met.

### Implementation

The principal investigator (PI) will supervise the randomization. Recruitment staff, who are responsible for initial evaluations, will begin this process. Next, the PI will return a digital form to the staff indicating the participant’s assigned group. The staff will then inform both the therapist and the family about the treatment details.

## Blinding

Due to the nature of the intervention, participants, parents, and therapists cannot be blinded to group allocation. However, the study’s specific hypotheses will be concealed from the caregivers and patients.

## Data collection and management

### Data collection methods

Questionnaire data from both the adolescents and the parents will be collected electronically using the online survey tool LimeSurvey [93]. The data will be exported from LimeSurvey by a designated study staff member and stored in a secure data network. The datasets will be organized by measurement time points and informant (adolescents or caregivers).

Data collected in paper-and-pencil form include the patient’s adherence, assessed using the clinician-rated Adherence Questionnaire as well as the patient-rated individual problem list and retrospective mood assessment. One designated study staff member will digitally input these data. Regular reviews of completeness, consistency, and plausibility will be conducted by two study staff members.

### Informants

In the JAY trial, informants will include the adolescent patient, their caregiver, and an unblinded clinician. Patients will undergo diagnostic sessions and cognitive-behavioural psychotherapy, and will use the JAY app to record mood and for diary activities. Furthermore, they will complete several self-report questionnaires at all measurement time points. The caregiver may be the biological parent or the child’s guardian. Caregivers will be involved in the study by participating in the in-person screening and information sessions along with the patients. They will complete caregiver-report questionnaires at all measurement time points. The unblinded clinician will be a study staff member who works in the area of diagnostics or therapy, or alternatively, a child and adolescent therapist from the Center for Child and Adolescent Cognitive Behavior Therapy (CEKIP). The unblinded clinician may also serve as the adolescent’s therapist which applies to both baseline (T0) and post-treatment (T3) assessments. The clinician will assess the patient’s symptoms based on information gained in the interviews during the diagnostic sessions.

### Retention

To promote participant retention and ensure complete follow-up, patients will receive email reminders to complete the follow-up questionnaire. If there is no response to the email, a reminder telephone call will be made. For participants who discontinue or deviate from the intervention protocols, any available outcome data collected up to that point will still be used in the analysis in order to maintain the integrity of the study results, as long as patient or parent consent is not withdrawn.

### Data management

Patients in the experimental group will have full control of their own data stored in the JAY smartphone app. They will decide which data they want to share, export them from the app, and upload them to a secure research server. From there, data will be stored in an encrypted data network provided and hosted by the University Hospital of Cologne.

Patients in the control group will hand their paper-based homework to their therapist, who will pass it to the project team. Again, adolescents can decide about data sharing and will have full control of their data. A designated study staff member will scan the homework and file the paper version in the respective patient file, which will be kept in a locked cabinet accessible only to study staff. The digital version will be stored in a secure network provided and hosted by the University Hospital of Cologne. Access to the project database will be restricted to authorized personnel of the research group only.

### Confidentiality

At the beginning of the study, each participant will be assigned a unique study identification number. All data collected during the study will be anonymized and can only be linked to the participant by the study staff, using the study identification number. Compliance with legal regulations for data protection will be ensured. Participating families will receive comprehensive information about their rights concerning data storage, protection, and deletion.

## Statistical methods

### Statistical methods for primary and secondary outcomes

#### Analyses of primary endpoint

The primary analysis set adheres to the intention-to-treat (ITT) principle, comprising all patients randomised with a valid baseline assessment. Additionally, per-protocol analyses will involve all patients who attend at least 50% of the planned treatment sessions and provide a valid follow-up. The primary endpoint is the overall THA score across the intervention (sessions 1–16). Subsequent analyses assess changes in phase 1 (sessions 1–8 from T1 to T2) and changes in phase 2 (sessions 9–16, between T2 and T3). A mixed model repeated measures multivariate analysis of variance will be conducted, with the between-subjects factor Group (smartphone-augmented vs. traditional), the within-subjects factor Time (T2, T3), and the interaction effect of Group × Time.

#### Analyses of secondary endpoints

For the secondary outcomes, we will conduct analyses utilising either mixed models for repeated measures, employing a heterogeneous first-order autoregressive structured covariance matrix over time, or generalized estimating equation approaches with corresponding marginal means and contrast tests (multilevel modelling). Time-to-dropout distributions will be summarised using the Kaplan-Meier method and compared using the (stratified) log-rank test. Efficacy variables will be summarised by time point and treatment arm, including mean, standard deviation, percentiles (minimum, 25th, 50th, 75th, and maximum), count, and percentage. Additionally, moderation, mediation, and conditional process modelling will be performed based on regression and structural equations (interaction, simple slope analysis; direct/indirect effects, kappa squared).

### Interim analyses

Interim analyses will be conducted with *N* = 18 patients per group to decide upon the continuation of the trial. If results are contrary to expectations, the PI will make a final decision regarding termination of the trial.

### Additional analyses

#### Patient demographics/other baseline characteristics

Demographic and baseline data will be collected at T0 and summarized descriptively for all documented patients. Continuous data will be summarized using measures such as arithmetic mean, standard deviation, minimum, 25th percentile, median, 75th percentile, maximum, and the number of complete and missing observations. If applicable, continuous variables may also be presented in a categorized form. Categorical data will be summarized according to the total number of patients in each category and the number of missing values. Relative frequencies will be expressed as valid percentages (number of patients divided by the number of patients with non-missing values).

#### Predictors/moderators of treatment outcome regarding anxiety and depressive symptoms

The following variables will be evaluated as potential moderators or predictors of treatment outcomes for internalizing symptoms at T3: (1) sociodemographic variables (e.g., number of children in the family; socioeconomic status and place of residence; caregivers’ age, education, income, and occupation), which will be assessed through a caregiver questionnaire at T0, (2) severity of comorbid symptoms using the self-report YSR/11-18R and the caregiver-report CBCL/6-18R at T0 and T3 (see above), (3) patients’ specific psychological characteristics, such as intelligence, measured at T0 using the CFT20-R (see above).

#### Mediators of change

Potential mediators that will be analyzed include (1) treatment fidelity as assessed by the therapist using an integrity questionnaire after each session. Therapists will document the interventions and treatment manuals that were used as well as the therapy homework the patient has to complete before the next session. (2) Additional potential mediators that will be analyzed are health-related quality of life, self-perception profiles, and emotion regulation strategies measured using the self-report KINDL^R^, Harter scale, and FEEL-KJ, respectively (see above). (3) Furthermore, treatment adherence assessed following each therapy session will be evaluated as a mediating factor using the Adherence Questionnaire (see above). This assessment will include four distinct items to define adherence, focusing on the patient’s engagement during the therapy sessions. Moreover, for each therapy-related homework task that the patient is given to complete before a session, execution adherence will be assessed after the session.

### Missing data

It is important to minimize treatment discontinuation among patients and ensure that all patients are followed up and documented after treatment discontinuation in order to collect the data according to the intention-to-treat (ITT) principle. Multiple imputation approaches will be employed to evaluate the potential impact of up to 20% attrition, considering proxy measures and assuming specific patterns of missingness not at random. These details will be documented in a statistical analysis plan. Additionally, supporting evidence will be provided by analyzing subjects who are essentially observed and treated per protocol.

## Oversight and monitoring

### Committees

This study is designed as a monocenter trial without external funding. Thus, there will be no external data monitoring or auditing committee. However, this will be considered for a subsequent larger trial.

### Harms

During each therapy session, therapists will inquire about potential adverse events, and participants will be encouraged to report any unexpected effects at any time during the study. All reported adverse events will be discussed with the PI in the regularly held supervision meetings. The principal investigator will be responsible for determining the necessary actions, including modifications to the protocol or, in extreme cases, terminating the trial.

## Discussion

The JAY trial aims to examine the effects of a newly developed psychotherapy app, used as an adjunct to a CBT treatment, on THA and therapy outcomes in 13–17-year-old adolescents with internalizing disorders. Effects on internalizing and comorbid symptoms as well as individually defined problems, quality of life, self-perception, emotion regulation strategies, and treatment satisfaction will be analyzed.

Previous research has mainly examined adult samples and has predominantly explored apps as substitutes for psychotherapy instead of CBT-adjacent apps. Moreover, effects of apps on THA have rarely been investigated. As such, this study will address a critical gap in the current knowledge regarding digital interventions for youth and their effects on THA, which might serve as a mediator of the effects on treatment outcomes when compared to traditional paper-and-pencil methods.

Several potential challenges that may arise during the JAY trial must be acknowledged. First, although it has already been discussed that most adolescents in Germany have their own smartphones [44] and the present-day youth is accustomed to living in an increasingly digital environment [43], technological barriers may arise. Some participants might not have a personal (smart-)phone or may be unfamiliar with the use of mobile applications. In the case of the former, loaner devices will be provided. These allow the mere use of the JAY app, and all other functionalities will be locked. Participants will receive an introductory session, in which the use of the app is explained. Staff members will be available for support if technological questions arise during therapy.

The app should be acceptable and user-friendly for adolescents, as otherwise, it could lead to low adherence, frustration, and disengagement among participants. In a smaller pilot study, the app was deployed in therapy sessions with adolescents and their therapists. In qualitative interviews, both parties reported that the app was user-friendly, intuitive, and appropriate for its purpose. It was noted that the introduction of a smartphone app altered the dynamics of the therapist-patient relationship, and patients perceived the app as a replacement for human interaction. This led patients to feel less connected to and supported by their therapist, and adherence to the therapy and the research protocol decreased. To overcome this problem, three sessions with the therapist were integrated into the study design, which precede the baseline phase and focus on building the foundation for a strong therapist-patient relationship and psychoeducation about the complementary role and benefits of the app.

Another important aspect to be considered is data privacy. To prevent a threat to confidentiality, apps should not make patients’ data available to third parties, and the transfer of data to the therapist should only take place with the patient’s informed consent [50, 56]. For this reason, participants will have full control over their data, which will be stored locally on their smartphones. Participants will decide themselves which data to share by uploading them to a secure research server.

Further methodological challenges include a potential bias due to the inability to blind patients and therapists to the interventions. In addition, the unblinded clinician responsible for the outcome assessment during the clinical interviews may also serve as the treating therapists, which could increase the risk of assessment bias. Participants will not be informed about the study hypotheses. Nevertheless, the information they receive may lead them to perceive the JAY app as innovative and anticipate higher adherence to therapy homework. Similarly, therapists may introduce bias when evaluating the therapy homework. In future studies, it is important to consider incorporating independent blinded evaluation of therapy homework adherence into the study design in order to mitigate potential biases.

A final aspect to consider concerns the highly effective nature of the control condition [49], which might make it difficult to identify the incremental effect of the app. It is possible that a larger sample will be required to detect significant differences.

Nonetheless, the JAY trial is expected to provide valuable insights into the applicability of a smartphone app used as an adjunct to CBT-based psychotherapy for youth, and into its impact on adherence to therapy homework between sessions. The app’s utility in supporting diagnostics and intervention will be evaluated, as well as its effect on various outcome measures. The results of this study will contribute to preparing research in the growing field of CBT app development and provide a scientific basis for the use of such apps in psychotherapy with adolescents.

Therefore, the findings of this study are expected to also have an impact on clinical practice and on the extension of treatment options for relevant mental disorders such as anxiety and depressive disorders.

### Public or patient involvement

The protocol was written based on the study design of AGD. There was no public or patient involvement in the planning of the trial or in the process of writing this study protocol.

### Trial status

Issue date: 08.08.2024.

Protocol amendment number: 03.

Authors: AGD, MD.

The recruitment for the JAY trial has started in October 2023 and is still ongoing at the time of submission of this protocol. The completion of recruitment is planned for March 2026.

## Supplementary Information

Additional file 1: Trial registration JAY.

Additional file 2: Model written informed consent.

Additional file 3: SPIRIT checklist.

## Data Availability

After the results are published, the final datasets can be obtained from the principal investigator (Anja Görtz-Dorten). The results of the study, especially regarding the primary outcome, will be submitted for publication in relevant journals. Furthermore, patients and their families will have access to the results if they wish. The results will also be presented at internal meetings and workshops as well as at relevant scientific congresses.

## Abbreviations

AUTHARK: App-augmented therapeutic treatment for children with aggressive behaviors
CBCL/6-18R: German version of the Revised Child Behavior Checklist for ages 6–18
CBT: Cognitive behavioral therapy
CEKIP: Center for Child and Adolescent Cognitive Behavior Therapy
CFT 20-R: German version of the Culture Fair Intelligence Test
DISYPS-III: Diagnostic System for Mental Disorders in Children and Adolescents
DISYPS-ILF: The comprehensive Structured Interview for Children and Adolescents according to ICD-10 and DSM-5 from the DISYPS Systems
DRKS: Deutsches Register Klinischer Studien (German Clinical Trials Register)
EMA: Ecological momentary assessment
FEEL-KJ: Modified version of the Questionnaire to Assess Children’s and Adolescents’ Emotion Regulation Strategies
ICC: Intraclass correlation coefficient
ICD-10: International Statistical Classification of Diseases and Related Health Problems, 10th revision
IPL: Individual problem list
ITT: Intention to treat
JAY: Journaling App for Youth
KINDL^R^: Revised Questionnaire for Health-Related Quality of Life Assessment in Children
PI: Principal investigator
RCT: Randomized controlled trial
THA: Therapy homework adherence
SCL-AD-P: Parent (or caregiver)-report Symptom Checklist for Anxiety Symptoms from the DISYPS-III
SCL-AD-S: Self-report Symptom Checklist for Anxiety Symptoms from the DISYPS-III
SCL-DEP-P: Parent (or caregiver)-report Symptom Checklist for Depressive Symptoms from the DISYPS-III
SCL-DEP-S: Self-report Symptom Checklist for Depressive Symptoms from the DISYPS-III
SUS: Modified German version of the System Usability Scale
YSR/11-18R: German version of the Revised Youth Self-Report for ages 11–18
ZUF-8-M: Modified version of the self-report Client Satisfaction Questionnaire
