# Supplementary material for: Effects of an app-augmented cognitive behavioral therapy using the Journaling App for Youth (JAY) for adolescents with internalizing disorders—a study protocol
Source: Trials. 2026 Jun 11;27:430. doi: 10.1186/s13063-026-09806-0 (PMC13255400; doi:10.1186/s13063-026-09806-0)
Supplement: Supplementary file 1 — Additional file 1: Trial registration JAY. [file 13063_2026_9806_MOESM1_ESM.pdf]

Vorschau der Studiendaten gedruckt am 21. February 2022 14:33:57

DRKS-ID der Studie: **DRKS00026623**

Studienbeschreibung

Titel der Studie

Wirksamkeit einer app-unterstützten verhaltenstherapeutischen Psychotherapie mit der Journaling App für Jugendliche (JAY) bei verschiedenen internalen psychischen Störungen

Studienakronym

JAY

Internetseite der Studie

https://akip.uk-koeln.de/ambulanzen/forschungsambulanz/fg-jay/

Allgemeinverständliche Kurzbeschreibung

Verhaltenstherapie ist bei den meisten psychischen Störungen das erste Mittel der Wahl. Die Wirksamkeit einer Verhaltenstherapie hängt unter anderem auch davon ab, inwiefern Inhalte aus dem Alltag der Patienten in die Therapiesitzung integriert- und andersherum die Inhalte aus der Therapie im Alltag umgesetzt werden. Neue Medientechnologien, wie Smartphone-Apps sind inzwischen auch im Alltag von vielen Kindern und Jugendlichen allgegenwärtig. Der Einsatz solcher Apps auf den ständig bei sich getragenen Smartphones kann bei der Umsetzung von therapeutischen Hausaufgaben im Alltag helfen und so die Wirksamkeit der Verhaltenstherapie weiter steigern.
In unserem Projekt wollen wir prüfen, ob und wie diese Therapien durch den unterstützenden Einsatz einer Smartphone-App noch zu verbessern sind. Neben den üblichen verhaltenstherapeutischen Methoden wird im Rahmen der Behandlung eine spezielle mobile Smartphone App mit dem Namen JAY (Journaling App for Youth) für den Altersbereich der 13- bis 17-Jährigen eingesetzt. Insgesamt durchlaufen die Jugendlichen 16 bis 24 wöchentliche Therapiesitzungen. Zusätzlich können 0-8 Sitzungen mit den Bezugspersonen erfolgen.

Folgende Fragen sollen im Verlauf der Studie beantwortet werden:
- Kann die Therapie-App bei internalen psychischen Störungen therapieunterstützend eingesetzt werden?
- Können durch die App-unterstütze Therapie die störungsspezifische Symptomatik und individuelle Probleme reduziert werden?
- Kann durch den Einsatz der Therapie-Apps die Umsetzung von therapeutischen Hausaufgaben verbessert werden?

Wissenschaftliche Kurzbeschreibung

Therapeutische Hausaufgaben sind ein wichtiger und einflussreicher Bestandteil der kognitiven Verhaltenstherapie (Kazantzis et al., 2010). Allerdings zeigen sich vor allem bei Kindern und Jugendlichen Motivationsdefizite diese therapeutischen Hausaufgaben umzusetzen (Petermann et al., 2012). Der Einsatz neuer Medientechnologie, wie Smartphone-Apps (Applikation, Anwendungssoftware für Mobilgeräte) kann die Umsetzung solcher Aufgaben erleichtern und die Motivation steigern (Tang & Kreindler, 2017). Insbesondere Smartphones sind im Alltag vieler Kinder und Jugendlicher allgegenwärtig (Medienpädagogischer Forschungsverbund Südwest, 2018, 2020) und ermöglichen so im therapeutischen Kontext neue Möglichkeiten den Transfer von in der Therapie erarbeiteten Inhalten in den Alltag zu verbessern und so die Effizienz der Verhaltenstherapie weiterhin zu steigern. Erste Überblicksarbeiten zu Smartphone-Apps für Kinder und Jugendliche mit psychischen Problemen weisen auf das Potenzial technikbasierter Interventionen hin (Domhardt et al., 2018), jedoch zeigen diese auch, dass es an Evidenz für deren Wirksamkeit mangelt (Grist et al., 2017). In dieser Studie soll die Wirksamkeit von App-unterstützter verhaltenstherapeutischer Psychotherapie bei Jugendlichen im Alter von 13 bis 17 Jahren

mit internalen (depressive Störungen, Angststörungen) psychischen Störungen in randomisierten Kontrollgruppendesign überprüft werden. Dafür wird neben den üblichen verhaltenstherapeutischen Methoden im Rahmen der Behandlung eine spezielle mobile Smartphone-App mit dem Namen JAY (Journaling App for Youth) eingesetzt.
Nach einer Einschlussmessung (T0) und Diagnostikphase erfolgt für alle Teilnehmenden die Randomisierung. Während der Diagnostikphase erfolgt bei allen Teilnehmenden über die JAY-App ("JAY-Journaling App for Youth") eine regelmäßige Abfrage der individuellen Probleme, erfasst über eine Verhaltensproblemliste, sowie eine Stimmungsabfrage bei den Jugendlichen.
Nach der Randomisierung werden in der Experimentalbedingung (N=35) bis zu 24 Sitzungen wöchentlicher Psychotherapie unter Einbezug der Therapie-App durchgeführt. Hierfür werden alle Funktionen der JAY-App für die PatientInnen aktiviert: Psychoedukationsfunktion, Momentary Assessment-Funktion, Videotagebuch-Funktion, Erinnerungsfunktion, Problemlösetrainingsfunktion, Bewältigungssklills-Funktion und Verstärkerfunktion.
In der Kontrollbedingung (N=35) werden die bis zu 24 Sitzungen wöchentlicher Psychotherapie ausschließlich über den Einsatz evidenzbasierter verhaltenstherapeutischer Methoden (Manualbasiert) durchgeführt. Zusätzlich können 2-6 Sitzungen mit den Bezugspersonen erfolgen. Im Rahmen der Studie sollen N=70 Jugendliche mit internalen psychischen Störungen (Angststörungen und depressive Störungen) einbezogen werden.

Zu insgesamt sechs Messzeitpunkten (inklusive Follow-Up sechs Monate nach Beendigung der Therapie) werden Daten im Selbst- und Fremdurteil erhoben.

Selbst vergebene Schlagwörter

Kognitive Verhaltenstherapie, Kinder- und Jugendlichenpsychotherapie, Smartphone gestützte Therapie, App-unterstützte Therapie, Randomisierte Kontrollgruppenstudie, RCT, internale Symptomatik, Angststörung, Depression

Planen Sie, die teilnehmerbezogene Daten anderen Forschern anonymisiert zur Verfügung zu stellen?

Nein

Beschreibung IPD sharing Plan:

[---]*

Organisatorische Daten

- DRKS-ID der Studie: **DRKS00026623**
- Registrierungsdatum im DRKS: **[---]***
- Registrierungsdatum im Partnerregister oder anderem Primärregister: **[---]***
- Wissenschaftsinitiierte Studie (IST/IIT): **ja**
- Antragsdatum bei der (federführenden) Ethikkommission: **01.10.2021**
- Datum des positiven Votums/der zustimmenden Bewertung der (federführenden) Ethikkommission: **27.01.2022**
- Votum der Ethikkommission: **Positives Votum/Zustimmende Bewertung**
- (federführende) Ethikkommissions Vorlage-Nr.: **21-1562, Ethik-Kommission der Medizinischen Fakultät der Universität zu Köln**

Sekundäre IDs

- Universal Trial Number (UTN): **U1111-1274-5475**

Untersuchte Krankheit/Gesundheitsproblem

- ICD10: **F32.0 - Leichte depressive Episode**
- ICD10: **F32.1 - Mittelgradige depressive Episode**
- ICD10: **F32.8 - Sonstige depressive Episoden**
- ICD10: **F32.9 - Depressive Episode, nicht näher bezeichnet**
- ICD10: **F40.0 - Agoraphobie**

- ICD10: **F40.00 - Agoraphobie: Ohne Angabe einer Panikstörung**
- ICD10: **F40.01 - Agoraphobie: Mit Panikstörung**
- ICD10: **F40.1 - Soziale Phobien**
- ICD10: **F40.8 - Sonstige phobische Störungen**
- ICD10: **F41.0 - Panikstörung [episodisch paroxysmale Angst]**
- ICD10: **F41.1 - Generalisierte Angststörung**
- ICD10: **F41.2 - Angst und depressive Störung, gemischt**
- ICD10: **F93.0 - Emotionale Störung mit Trennungsangst des Kindesalters**
- ICD10: **F93.2 - Störung mit sozialer Ängstlichkeit des Kindesalters**
- ICD10: **F43.2 - Anpassungsstörungen**

Interventionsgruppen/Beobachtungsgruppen

- Arm 1: **In der Experimentalbedingung (N=35) werden bis zu 24 Sitzungen wöchentlicher Psychotherapie unter Einbezug der Therapie-App durchgeführt. Hierfür werden alle Funktionen der JAY-App für die PatientInnen aktiviert: Psychoedukationsfunktion, Momentary Assessment-Funktion, Videotagebuch-Funktion, Erinnerungsfunktion, Problemlösetrainingsfunktion, Bewältigungssklills-Funktion und Verstärkerfunktion. Zusätzlich können 2-6 Sitzungen mit den Bezugspersonen erfolgen.**
- Arm 2: **In der Kontrollbedingung (N=35) werden die bis zu 24 Sitzungen wöchentlicher Psychotherapie ausschließlich über den Einsatz evidenzbasierter verhaltenstherapeutischer Methoden (Manualbasiert) durchgeführt. Zusätzlich können 2-6 Sitzungen mit den Bezugspersonen erfolgen.**

Charakteristika

- Studientyp: **Interventionell**
- Studientyp nicht-interventionell: **[---]***
- Studiendesign Zuteilung: **Kontrollierte, randomisierte Studie**
- Verblindung: **Offen**
- Wer ist verblindet: **[---]***
- Kontrolle: **Aktive Kontrolle (wirksame Behandlung der Kontrollgruppe)**
- Studienzweck: **Therapie**
- Gruppenzuteilung: **Parallelverteilung**
- Studienphase: **II**
- Off-label use (Zulassungsüberschreitende Anwendung eines Arzneimittels): **Nicht zutreffend**

Primärer Endpunkt

Als primäre Zielgröße der RCT-Studie wird die Therapieaufgaben-Adhärenz über den Adhärenzbogen zu Therapieaufgaben (Therapeuten) erfasst.

Sekundärer Endpunkt

Als sekundäre Zielgrößen werden die Veränderung der komorbiden Symptomatik und des Funktionsniveaus
der Jugendlichen im Eltern- und Selbsturteil erhoben. Weitere Zielgrößen werden erfasst: Anzahl der Therapiesitzungen/ Therapiedauer, Zufriedenheit der Patienten, Therapieaufgaben-Adhärenz (Selbsturteil), Therapie-Adhärenz insgesamt (klinisches Urteil), Therapieerfolg.

Länder in denen Studienteilnehmer rekrutiert werden

- DE: **Deutschland**

Rekrutierungsstandorte

- **Klinik** ☐ Universitätsklinikum Ausbildungsambulanz für Kinder- und Jugendlichenpsychotherapie der Uniklinik Köln, Köln

Rekrutierung

- Geplant/Tatsächlich: **Geplant**

- (geplantes/tatsächliches Datum) Einschluss des ersten Studienteilnehmers: **01.03.2022**
- Geplante Studienteilnehmeranzahl gesamt: **70**
- Monozentrisch/Multizentrisch: **Monozentrisch**
- National/International: **National**

Einschlusskriterien

- Geschlecht: **Beide, männlich und weiblich**
- Mindestalter: **13 Jahre**
- Höchstalter: **17 Jahre**

Weitere Einschlusskriterien

Einschlusskriterium ist die klinische Diagnose einer der folgenden Störungen nach ICD-10:
- depressive Episoden (F32.0, F32.1, F32.8, F32.9)
- Angststörungen (F40.0 (F40.00, F40.01), F40.1, F40.8, F41.0, F41.1, F41.2)
- Emotionale Störungen des Kindesalters (F93.0, F93.2)
- Anpassungsstörung (F43.2)

Weiteres Einschlusskriterium ist ein auffälliger Wert im Selbsturteil (YSR, Achenbach & Rescorla 2001) für die Skala Internal (d.h. T-Wert ≥ 60) sowie mindestens ein bestehendes Problem in der Verhaltensproblemliste des/ der Jugendlichen (d.h. Wert ≥3). Zudem sollte die Familie über ausreichende Deutschkenntnisse verfügen.

Ausschlusskriterien

Ausschlusskriterien sind die Diagnose einer Tiefgreifenden Entwicklungsstörung (ICD-10: F84), Spezifische
Phobie (ICD-10: F40.2) oder einer anderen psychischen Störung, die im Vordergrund steht, ein IQ unter 80 (CFT 20-R, Weiß, 2006; CFT 1-R, Weiß & Osterland, 2012) und krisenhafte Zuspitzungen, die eine stationäre Behandlung nahelegen. Parallel darf keine weitere Psychotherapie in Anspruch genommen werden. Ferner soll die Indikation für den Neubeginn einer Pharmakotherapie bzw. für eine Dosierungsveränderung einer bestehenden Pharmakotherapie zum Zeitpunkt der Intervention ausgeschlossen
werden.

Adressen

Primärer Sponsor

Ausbildungsinstitut für Kinder- und Jugendlichenpsychotherapie der Uniklinik Köln (Akip)
Pohligstraße 9
50969 Köln
Deutschland
Telefon: **[---]***
Fax: **[---]***
E-Mail: **[---]***
URL der Einrichtung: **[---]***

Kontakt für wissenschaftliche Anfragen

Ausbildungsinstitut für Kinder- und Jugendlichenpsychotherapie der Uniklinik Köln Evaluation/ Forschungsbereich Psychotherapie Kölner Institut der Christoph-Dornier-Stiftung für Klinische Kinderpsychologie
Frau Prof. Dr. rer medic. Anja Görtz-Dorten
Pohligstr. 9
50969 Köln
Deutschland
Telefon: **0221 478-76836**
Fax: **[---]***
E-Mail: **anja.goertz-dorten at uk-koeln.de**

URL der Einrichtung: **[---]***

Kontakt für Studienteilnehmer
AKiP Köln
Frau
Pohligstraße 9
50969 Köln
Deutschland
Telefon: **0221 478 87772**
Fax: **[---]***
E-Mail: **jay-forschung at uk-koeln.de**
URL der Einrichtung: **https://akip.uk-koeln.de/ambulanzen/forschungsambulanz/fg-jay/**

Finanzierungsquellen

Haushaltsmittel, keine fremden Finanzmittel (Budget des Studienleiters)
Ausbildungsinstitut für Kinder- und Jugendlichenpsychotherapie der Uniklinik Köln (Akip)
Pohligstraße 9
50969 Köln
Deutschland
Telefon: **[---]***
Fax: **[---]***
E-Mail: **[---]***
URL der Einrichtung: **[---]***

Status

- Status der Rekrutierung: **Rekrutierung geplant**
- Grund, falls "Status der Rekrutierung" "permanent eingestellt" oder "zurückgezogen": **[---]***
- Grund, falls Grund des Rekrutierungsstopps "Anderer": **[---]***
- Tatsächliches Datum des Studienabschlusses (LPLV): **[---]***
- Tatsächliche Gesamtzahl Studienteilnehmer in Deutschland nach abgeschlossener Rekrutierung: **[---]***
- Tatsächliche Gesamtzahl Studienteilnehmer aller Zentren nach abgeschlossener Rekrutierung: **[---]***

Publikationen, Studienergebnisse und weitere Studiendokumente

- **[---]***

Alle grau hinterlegten Felder werden in der öffentlichen Ansicht nicht angezeigt.
** Dieser Eintrag bedeutet, dass der Parameter entweder nicht zutrifft oder dass er nicht eingetragen wurde.*

Vorschau der Studiendaten gedruckt am 21. February 2022 14:33:57

DRKS-ID: **DRKS00026623**

Trial Description

Title

Effectiveness of an app-assisted cognitive behaviour therapy by use of the Journaling App for Youth (JAY) for different internalising disorders

Trial Acronym

JAY

URL of the Trial

https://akip.uk-koeln.de/ambulanzen/forschungsambulanz/fg-jay/

Brief Summary in Lay Language

Cognitive behavioural therapy (CBT) is the first choice for treatment of most of the mental disorders. The effectiveness of CBT also depends on how well content from the patient's daily life can be integrated into therapy and how well content from therapy can be implemented into daily life. New media technologies such as smartphone apps are already ubiquitous in the daily life of many children and adolescents. The usage of those apps on personal devices which are always carried around with oneself could help with the implementation of therapeutic homework in daily life and therefore increase the effectiveness of CBT. In our project, we aim to investigate if and how therapy can be improved by use of an assisting smartphone app. Next to conventional CBT methods, a special smartphone app named JAY (Journaling App for Youth) for adolescents from age 13 to 17 years will be integrated into therapy. Overall, participating adolescents receive between 16 and 24 weekly therapy sessions. Up to eight sessions with caregivers might take place. The following questions are to be answered during the study: - Can the therapy app assist in therapy for internalising mental disorders? - Can disorder-specific symptomology and individual problems be reduced by app-assisted therapy? - Can implementation of therapeutic homework be improved by use of therapy apps?

Brief Summary in Scientific Language

Therapeutic homework is an important and influential part of cognitive behavior therapy (Kazantzis et al., 2010). However, especially in children and adolescents, there is a lack of motivation to do this therapeutic homework (Petermann et al., 2012). The use of new media technology, such as smartphone apps (applications, application software for mobile devices) can facilitate the implementation of such tasks and increase motivation (Tang & Kreindler, 2017). Smartphones, in particular, are ubiquitous in the everyday life of many children and adolescents (Medienpädagogischer Forschungsverbund Südwest, 2018, 2020) and thus enable new possibilities in the therapeutic context to improve the transfer of content developed in therapy into everyday life and thus to further increase the efficiency of behavioral therapy. Initial reviews of smartphone apps for children and adolescents with mental health problems indicate the potential of technology-based interventions (Domhardt et al., 2018), but these also show that there is a lack of evidence for their effectiveness (Grist et al., 2017) . The aim of this study is to examine the effectiveness of app-supported behavioral psychotherapy in adolescents aged 13 to 17 years with internal (depressive disorders, anxiety disorders) mental disorders in a randomized control group design. In addition to the usual behavioral therapy methods, a special mobile smartphone app called JAY (Journaling App for Youth) is used as part of the treatment.
After an inclusion measurement (T0) and a diagnostic phase, all participants are randomized. During the diagnostic phase, all participants are regularly queried via the JAY app ("JAY Journaling App for Youth") about their individual problems, recorded using a list of behavioral problems, as well as a questionnaire on the young people's mood.
After randomization, up to 24 sessions of weekly psychotherapy are carried out in the experimental condition (N = 35) using the therapy app. For this purpose, all functions of the JAY app are activated for the patients: psychoeducational function, momentary assessment function, video diary function, reminder function, problem-solving training function, coping skills function and reinforcement function.
In the control condition (N = 35), the up to 24 sessions of weekly psychotherapy are carried out solely using evidence-based behavioral therapy methods (manual-based). In addition, 2-6 caregiver sessions can take place. In total, N = 70 adolescents with internal mental disorders (anxiety disorders and depressive disorders) are to be included in the study.

At a total of six measurement time points (including a follow-up six months after the end of the therapy), data is collected in self and other reports.

Keywords

Cognitive behaviour therapy, child and adolescent psychotherapy, smarthphone-assisted therapy, app-assisted therapy, randomised controlled trial, RCT, interalising symptoms, anxiety disorder, depression

Do you plan to share individual participant data with other researchers?

No

Description IPD sharing plan:

[---]*

Organizational Data

- DRKS-ID: **DRKS00026623**
- Date of Registration in DRKS: **[---]***
- Date of Registration in Partner Registry or other Primary Registry: **[---]***
- Investigator Sponsored/Initiated Trial (IST/IIT): **yes**
- Date of (leading) Ethics Committee Application: **2021/10/01**
- Date of (leading) Ethics Committee Approval: **2022/01/27**
- Ethics Approval/Approval of the Ethics Committee: **Approved**
- (leading) Ethics Committee No.: **21-1562, Ethik-Kommission der Medizinischen Fakultät der Universität zu Köln**

Secondary IDs

- Universal Trial Number (UTN): **U1111-1274-5475**

Health Condition or Problem studied

- ICD10: **F32.0 - Mild depressive episode**
- ICD10: **F32.1 - Moderate depressive episode**
- ICD10: **F32.8 - Other depressive episodes**
- ICD10: **F32.9 - Depressive episode, unspecified**
- ICD10: **F40.0 - Agoraphobia**
- ICD10: **F40.00 - [generalization F40.0: Agoraphobia]**
- ICD10: **F40.01 - [generalization F40.0: Agoraphobia]**
- ICD10: **F40.1 - Social phobias**
- ICD10: **F40.8 - Other phobic anxiety disorders**
- ICD10: **F41.0 - Panic disorder [episodic paroxysmal anxiety]**
- ICD10: **F41.1 - Generalized anxiety disorder**
- ICD10: **F41.2 - Mixed anxiety and depressive disorder**
- ICD10: **F93.0 - Separation anxiety disorder of childhood**
- ICD10: **F93.2 - Social anxiety disorder of childhood**
- ICD10: **F43.2 - Adjustment disorders**

Interventions/Observational Groups

- Arm 1: **In the experimental condition (N=35), up to 24 sessions of weekly psychotherapy are carried out using the therapy app. For this purpose, all functions of the JAY app are activated for the patients: psychoeducational function, momentary assessment function, video diary function, reminder function, problem-solving training function, coping skills function and reinforcement function. In addition, 2-6 caregiver sessions can take place.**
- Arm 2: **In the control condition (N = 35), the up to 24 sessions of weekly psychotherapy are carried out solely using evidence-based behavioral therapy methods (manual-based). In addition, 2-6 caregiver sessions can take place.**

Characteristics

- Study Type: **Interventional**
- Study Type Non-Interventional: **[---]***
- Allocation: **Randomized controlled trial**
- Blinding: **Open (masking not used)**
- Who is blinded: **[---]***
- Control: **Active control (effective treament of control group)**
- Purpose: **Treatment**
- Assignment: **Parallel**
- Phase: **II**

- Off-label Drug use: **N/A**

Primary Outcome

Primary outcome of the RCT is adherence to therapy exercises which is recorded on the adherence sheet to therapy homework (therapist).

Secondary Outcome

Secondary outcomes are the change in comorbid symptomology and level of functioning of the adolescent based on self- and parent-report. Other outcomes that are measured: number of therapy sessions/duration of therapy, satisfaction of the patient, adherence to therapy homework (self-report), adherence to therapy overall (clinical judgment), therapy success.

Countries of Recruitment

- DE: **Germany**

Locations of Recruitment

- **Medical Center** ☐ Universitätsklinikum Ausbildungsambulanz für Kinder- und Jugendlichenpsychotherapie der Uniklinik Köln, Köln

Recruitment

- Planned/Actual: **Planned**
- (Anticipated or Actual) Date of First Enrollment: **2022/03/01**
- Target Sample Size: **70**
- Monocenter/Multicenter trial: **Monocenter trial**
- National/International: **National**

Inclusion Criteria

- Gender: **Both, male and female**
- Minimum Age: **13 Years**
- Maximum Age: **17 Years**

Additional Inclusion Criteria

Inclusion criterion is the clinical diagnosis of one of the following disorders according to ICD-10:
- depressive episodes (F32.0, F32.1, F32.8, F32.9)
- Anxiety disorders (F40.0 (F40.00, F40.01), F40.1, F40.8, F41.0, F41.1, F41.2)
- childhood emotional disorders (F93.0, F93.2)
Adjustment disorder (F43.2)

Another inclusion criterion is a clinically relevant score on the Internal scale (i.e. T value ≥ 60) of the self-report (YSR, Achenbach & Rescorla 2001) as well as at least one existing problem in the adolescent's behaviour problem list (i.e. value ≥3 at T0; mean value ≥ 3 at T1 over the last three measurements in the diagnostic phase). In addition, the family should have sufficient German language skills.

Exclusion Criteria

Exclusion criteria are the diagnosis of a pervasive developmental disorder (ICD-10: F84), specific phobia (ICD-10: F40.2) or any other mental health disorder that is predominant, an IQ below 80 (CFT 20-R, Weiß, 2006; CFT 1-R, Weiß & Osterland, 2012) and critical escalations that suggest inpatient treatment. Participants cannot receive further psychotherapy at the same time. Furthermore, the indication for a new start of pharmacotherapy or for a change in the dosage of an existing pharmacotherapy at the time of the intervention should be excluded.

Addresses

Primary Sponsor

Ausbildungsinstitut für Kinder- und Jugendlichenpsychotherapie der Uniklinik Köln (Akip)
Pohligstraße 9
50969 Köln
Germany
Telephone: **[---]***
Fax: **[---]***
E-mail: **[---]***
URL: [---]*

Contact for Scientific Queries

Ausbildungsinstitut für Kinder- und Jugendlichenpsychotherapie der Uniklinik Köln Evaluation/ Forschungsbereich Psychotherapie Kölner Institut der Christoph-Dornier-Stiftung für Klinische Kinderpsychologie
Ms. Prof. Dr. rer medic. Anja Görtz-Dorten
Pohligstr. 9
50969 Köln
Germany
Telephone: **0221 478-76836**
Fax: **[---]***
E-mail: **anja.goertz-dorten at uk-koeln.de**
URL: [---]*

Contact for Public Queries

AKiP Köln
Ms.
Pohligstraße 9
50969 Köln
Germany
Telephone: **0221 478 87772**
Fax: **[---]***
E-mail: **jay-forschung at uk-koeln.de**
URL: https://akip.uk-koeln.de/ambulanzen/forschungsambulanz/fg-jay/

Sources of Monetary or Material Support

Institutional budget, no external funding (budget of sponsor/PI)

Ausbildungsinstitut für Kinder- und Jugendlichenpsychotherapie der Uniklinik Köln (Akip)
Pohligstraße 9
50969 Köln
Germany
Telephone: **[---]***
Fax: **[---]***
E-mail: **[---]***
URL: [---]*

Status

- Recruitment Status: **Recruiting planned**
- Reason, if "Recruitment stopped after recruiting started" or "Recruiting withdrawn before recruting started": **[---]***
- Reason, if Reason for Recruiting Stop "Other": **[---]***
- Study Closing (LPLV): **[---]***
- Number of Participants in Germany after Recruiting complete: **[---]***
- Total Number of Participants (all Sites worldwide) after Recruiting complete: **[---]***

Trial Publications, Results and other Documents

- **[---]***

All grey highlighted fields will not be displayed on the public web site.
** This entry means the parameter is not applicable or has not been set.*
