# Supplementary material for: Effects of an app-augmented cognitive behavioral therapy using the Journaling App for Youth (JAY) for adolescents with internalizing disorders—a study protocol
Source: Trials. 2026 Jun 11;27:430. doi: 10.1186/s13063-026-09806-0 (PMC13255400; doi:10.1186/s13063-026-09806-0)
Supplement: Supplementary file 2 — Additional file 2: Model written informed consent. [file 13063_2026_9806_MOESM2_ESM.pdf]

Teilnehmendeninformation für Jugendliche über die Teilnahme an der Studie:

Evaluation einer therapieunterstützenden Smartphone-App für Jugendliche (JAY) bei internalen psychischen Störungen.

Hallo,

wir freuen uns, dass du an unserer Forschung zu der in unserem Haus entwickelten, therapieunterstützenden Smartphone-App JAY (Journaling App for Youth) interessiert bist und uns durch deine Teilnahme hierbei unterstützen möchtest. Im Folgenden möchten wir dich über die Ziele und den Verlauf der Studie informieren und dir erklären, warum deine Mitarbeit im Falle einer Studienteilnahme wichtig ist. Die Studie wird zu Forschungszwecken durchgeführt.

Wir bitten dich, diese Information sorgfältig zu lesen und anschließend zu entscheiden, ob du an dieser Studie teilnehmen möchtest oder nicht.

Die Teilnahme an dieser Studie ist freiwillig. Du wirst in diese Studie also nur dann einbezogen, wenn du dazu schriftlich deine Einwilligung erklärst. Sofern du nicht an der Studie teilnehmen willst oder später deine Einwilligung widerrufst, entstehen für dich daraus keine Nachteile.

Ein*e Studienmitarbeiter*in hat dir bereits eine Reihe von Informationen zu der geplanten Studie gegeben. Der nachfolgende Text soll dich über die wichtigen Aspekte der Studie, insbesondere die Ziele und den Ablauf informieren. Lies die Teilnehmendeninformation bitte sorgfältig und gewissenhaft. Anschließend wird ein*e Studienmitarbeiter*in das Aufklärungsgespräch mit dir führen. Bitte zögere nicht, alle Punkte anzusprechen, die dir unklar sind. Du wirst danach ausreichend Bedenkzeit erhalten, um über deine Teilnahme zu entscheiden.

*Auftraggebende der Studie und Studienleiter*in*

Ausbildungsinstitut für Kinder- und Jugendlichenpsychotherapie an der Uniklinik Köln (AKiP Köln)
Klinik und Poliklinik für Psychiatrie, Psychosomatik und Psychotherapie des Kindes- und Jugendalters an der Uniklinik Köln
Prof. Dr. Anja Görtz-Dorten
Prof. Dr. Manfred Döpfner

1. Ziel der Studie

In dieser Studie soll die Wirksamkeit von App-unterstützter verhaltenstherapeutischer Psychotherapie bei Jugendlichen, die Angst haben und/ oder häufig traurig sind, überprüft werden. Dafür wird neben den üblichen verhaltenstherapeutischen Methoden im Rahmen der Behandlung für eine Gruppe der Jugendlichen eine spezielle mobile Smartphone-App mit dem Namen JAY (Journaling App for Youth) für den Altersbereich der 13-17-Jährigen eingesetzt. Die App enthält Funktionen, die die Übertragung von Therapieinhalten in den Alltag erleichtern, aber auch Alltagssituationen für die Therapie leichter zugänglich machen sollen. Zu diesen Funktionen zählen das Führen eines digitalen Tagebuchs (per Video oder schriftlicher Eingabe), eine

Stimmungsabfrage-Funktion, bei der zu definierten Zeitpunkten die aktuellen Gefühle abgefragt werden, die Erinnerung an individuell vereinbarte Therapieaufgaben, eine Trainingsfunktion der erarbeiteten Inhalte, sowie eine Funktion, über die verschiedene Strategien zur Bewältigung von schwierigen Situationen vorgegeben werden (Bewältigungsskills-Funktion).

2. Art der Studie

Die Wirksamkeit, Anwendbarkeit und Zufriedenheit von App-unterstützter Psychotherapie sollen bei Jugendlichen, die sich in der Psychotherapieambulanz des AKiP Köln vorstellen, überprüft werden.

An der Studie nehmen insgesamt 70 Jugendliche zwischen 13 und 17 Jahren mit verschiedenen internalen psychischen Störungen teil.

Zunächst durchlaufen alle interessierten Jugendlichen und ggf. deren Bezugspersonen eine ausführliche Eingangsuntersuchung. Hierzu werden eine Reihe von Fragebögen ausgegeben und persönliche Interviews geführt. In diesem Kontext besprechen wir, ob es sinnvoll und für dich in Ordnung ist, deine Eltern und ggf. bei Bedarf deine Lehrer*innen in die Behandlung miteinzubeziehen. Mit deiner Erlaubnis geben wir auch Fragebögen für deine Eltern und/oder Lehrer*innen raus. Im Gegensatz zu einer Behandlung außerhalb der Studie sind diese ersten Befragungen ausführlicher.

Die Wirksamkeit von App-unterstützter verhaltenstherapeutischer Psychotherapie wird im direkten Vergleich mit einer Behandlung, in der nur mit papierbasierten Materialien ohne App-Einsatz verhaltenstherapeutisch gearbeitet wird, erfasst. Um andere Einflüsse auf die Wirksamkeit so gering wie möglich zu halten, werden die Jugendlichen nach dem Zufallsprinzip in zwei gleich große Gruppen aufgeteilt.

Die eine Hälfte der Jugendlichen (50%) erhält verhaltenstherapeutische Psychotherapie in Kombination mit der therapieunterstützenden App JAY (App-Gruppe).

Die andere Hälfte der Jugendlichen (50%) erhält verhaltenstherapeutische Psychotherapie (Papier-Gruppe).

Die JAY-App kannst du dir kostenlos auf dein eigenes Smartphone herunterladen oder du bekommst ein Smartphone von uns zur Verfügung gestellt. Das Leihhandy von uns ist ausschließlich zur Verwendung der App eingestellt, alle anderen Funktionen sind gesperrt.

3. Ablauf der Studie

Wenn du dich für eine Studienteilnahme entscheidest, folgen zunächst 8 Termine, in denen wir die Diagnostik durchführen werden und in denen du die Studie, die App und deine*n Therapeut*in kennenlernen wirst. Wir planen für diese Termine einen Zeitraum von ca. 4 Wochen ein. Dann folgt eine Phase der Selbstbeobachtung von 4 Wochen, in der du deine Stimmung beobachtest und dokumentierst. In dieser Zeit finden keine Therapietermine statt. Darauffolgend werden bis zu 24 Sitzungen wöchentlicher ambulanter Psychotherapie unter Einbezug der Therapie-App oder ohne die App stattfinden. Bei Bedarf können zusätzlich Termine mit deinen Bezugspersonen durchgeführt werden. Eine Stimmungsabfrage erfolgt mehrmals täglich in der Selbstbeobachtungsphase und zwischen bestimmten Therapiesitzungen. Weiterhin beinhalten Therapiehausaufgaben zweimal wöchentlich das Führen des Tagebuchs sowie eine individuell abgesprochene Aufgabe. In der App-Gruppe werden diese Aufgaben mithilfe der entsprechenden App-Funktionen erfüllt, in der Papier-Gruppe werden entsprechende Papierbogen ausgegeben. Die weiteren App-Funktionen werden individuell nach Absprache mit den Behandelnden eingesetzt. Die therapeutischen Inhalte werden entsprechend aktueller wissenschaftlicher Erkenntnisse ausgewählt und orientieren sich an Manualen, die unter anderem bei AKiP Köln entwickelt und bereits untersucht wurden.

Zu unseren Messzeitpunkten T2, T3 und T4 (siehe Abbildung) bzw. nach 8, 16 und 24 Sitzungen, erfolgen weitere Befragungen per Fragebogen. Zu T3 wird außerdem erneut ein Interview geführt.

Nach der 16. Sitzung werden wir dann gemeinsam prüfen, ob weitere 8 Therapiesitzungen im Rahmen der Studie erfolgen. Wenn die angebotenen Behandlungsmaßnahmen nach diesen weiteren 8 Sitzungen nicht den gewünschten Erfolg zeigen, werden wir nach Abschluss der Untersuchung mit dir überlegen, welche anderen Hilfen wir anbieten oder andere Einrichtungen wir empfehlen können. Weitere 6 Monate nach Studienende erfolgt eine letzte Befragung.

Falls im Laufe der Behandlung deutlich wird, dass alternative oder intensivere therapeutische Maßnahmen notwendig sind, kann die Teilnahme an der Studie jederzeit beendet werden.

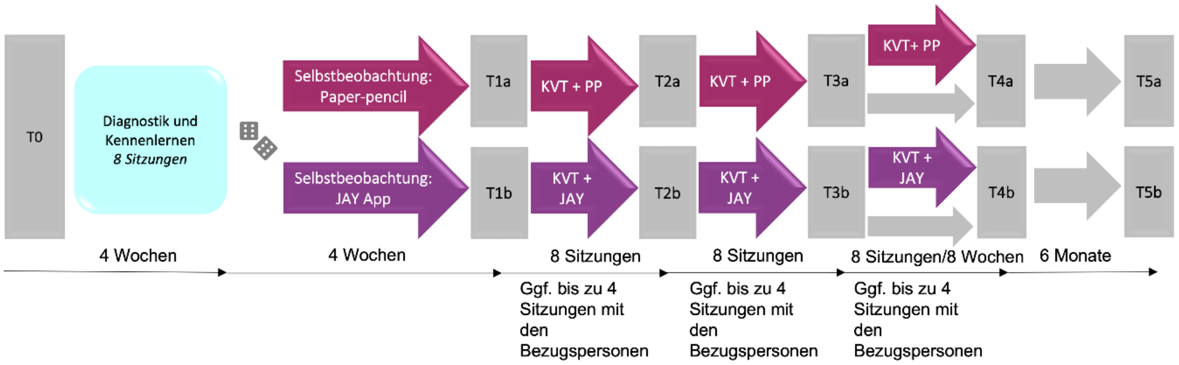

Die Ethikkommission der Medizinischen Fakultät zu Köln hat über das vorliegende Forschungsvorhaben beraten und zustimmend bewertet.

Die klinische Prüfung der App-unterstützten Psychotherapie wird aus Eigenmitteln des Ausbildungsinstituts für Kinder- und Jugendlichenpsychotherapie (AKiP Köln) an der Uniklinik Köln finanziert.

4. Wer kann an der Studie teilnehmen?

Teilnehmen können Jugendliche, die zwischen 13 und 17 Jahre alt sind und Schwierigkeiten in den folgenden Bereichen haben: häufiges Traurigsein (depressive Störungen) und/ oder starke Angst (Angststörungen). Die Einordnung und Beurteilung des Problemverhaltens findet auf Basis deiner Informationen vor Studienbeginn durch die Studienmitarbeitenden statt. Diese Informationen werden mithilfe von Interviews und Fragebögen von dir und deinen Eltern erhoben. Wenn du nicht möchtest, dass deine Eltern mit in die Behandlung einbezogen werden, dann ist das vollkommen in Ordnung. Du musst dein schriftliches Einverständnis zur Teilnahme an der Studie geben und mit dem App-Einsatz einverstanden sein.

Nicht teilnehmen können Personen mit der Diagnose einer tiefgreifenden Entwicklungsstörung (Einschätzung durch Studienmitarbeitenden), für die keine kognitive Verhaltenstherapie angezeigt ist, die eine stationäre Behandlung benötigen, die eine weitere Psychotherapie in Anspruch nehmen, bei denen voraussichtlich ein Neubeginn/ eine Änderung der Medikation notwendig ist (Einschätzung durch Studienmitarbeitenden) und die nicht schriftlich eingewilligt haben.

Im Gesprächstermin klären wir gemeinsam ab, ob es sinnvoll ist, dass du an der Studie teilnimmst.

Die geplante Gesamt-Laufzeit dieses Vorhabens entspricht ca. einem Jahr. Es sollen insgesamt 70 Jugendliche an der Studie teilnehmen.

5. Abwägung von möglichem Nutzen und Risiko der Studie

Wir können nicht mit Sicherheit sagen, ob du von der Studienteilnahme profitieren wirst. Alle durch uns angebotenen Hilfen entsprechen den aktuellen wissenschaftlichen Erkenntnissen. Wir erhoffen

uns jedoch einen Nutzen für die Wissenschaft und die Verbesserung der Versorgung und Behandlung von Kindern und Jugendlichen mit psychischen Störungen.

Die häufige, exzessive und missbräuchliche Nutzung von „Smartdevices“ (Smartphone, Tablet) kann möglicherweise Risiken beinhalten. Während der Teilnahme an unserer Studie wirst du jedoch die Smartphone App im Rahmen weniger Wochen, täglich nur für einen kurzen Zeitraum nutzen. Bei den Leihandys sind alle weiteren Funktionen des Gerätes zudem deaktiviert, so dass der Anreiz für eine längere Nutzung sehr gering ist. Für einen vergleichsweise kurzen Nutzungszeitraum sind uns keine Risiken bekannt. Vor der selbständigen Nutzung im Alltag wird mit dir genau besprochen, wie die Therapie-App anzuwenden ist, welche Art von Daten dadurch erfasst werden und welche Folgen sich daraus für den Umgang mit dem Smartphone ergeben. Zum Schutz der Daten vor unautorisierten Personen wird das Handy durch ein Passwort geschützt.

6. Geplante Maßnahmen und Ablauf der Teilnahme

Falls du dich für die Teilnahme an der Studie entscheidest, erhältst du wöchentliche, ambulante Verhaltenstherapie, wie sie üblicherweise auch außerhalb von Studien in Regeltherapien durchgeführt wird. Zusätzlich wirst du die Therapie-App oder die schriftliche Dokumentation entsprechend individueller Absprache zwischen den Therapiesitzungen einsetzen. Der zeitliche Rahmen, in welchem die App verwendet wird, ist genau definiert und entspricht in etwa dem von regulären, üblicherweise handschriftlich ausgefüllten therapeutischen Hausaufgaben, sodass der Einsatz nicht als zusätzliche Belastung angesehen wird.

Das bedeutet, dass du in bestimmten Wochen mehrmals täglich über die App nach deiner Stimmung gefragt wirst (ca. 1 Minute) und ab Therapiebeginn zweimal wöchentlich ein Tagebuch führst (ca. 5 Minuten) und die Durchführung deiner Therapiehausaufgabe dokumentierst (ca. 2 Minuten). Die Erhebung von Fragebögen (6 Mal im Rahmen der Studie) und Interviews (2 Mal innerhalb der Studie) erfolgt häufiger als in der Routineversorgung üblich.

Über gesundheitliche Veränderungen jeglicher Art sind die Behandelnden umgehend zu informieren.

Insgesamt wird deine individuelle Teilnahme an der Studie circa 6 Monate (zusätzlich eine Messung sechs Monate nach Ende der Behandlung) dauern. Die Sitzungen finden einmal wöchentlich statt und dauern je 50 Minuten.

7. Alternative Behandlungsmethoden

Du musst nicht an dieser Studie teilnehmen, wenn du das nicht willst. Falls du dich gegen eine Studienteilnahme entscheidest, werden wir dich zu weiteren Unterstützungsangeboten beraten (z.B. Erziehungsberatungsstelle oder Anbindung in einer kinder- und jugendpsychiatrischen Praxis). Alternativ kann eine Behandlung bei AKiP Köln auch ohne Studienteilnahme stattfinden.

8. Datenverarbeitung und Datenschutz

Während der Studie werden Informationen von dir erhoben, niedergeschrieben und elektronisch gespeichert. Hierbei handelt es sich um personenbezogene Daten einer besonderen Kategorie (über die Gesundheit, Geschlecht, Alter usw.). Im Rahmen der Studie werden diese Daten/Krankheitsdaten (z. B. Alter, Geschlecht) pseudonymisiert, das heißt ohne Namensnennung, sondern nur codiert durch z. B. eine Nummer abgespeichert. Eine Zuordnung ist nur über eine bei den Behandelnden hinterlegte Identifikationsliste möglich.

Die Rechtsgrundlage für die Datenverarbeitung ist deine freiwillige, schriftliche Einwilligung (Art. 6 DSGVO). Die im Rahmen der Studie erhobenen Daten werden auf dem V-Laufwerk der Uniklinik Köln in einem zugangsbeschränkten Ordner gespeichert, auf den nur autorisierte Mitarbeitende im Netzwerk der Uniklinik Köln Zugriff haben. Im Anschluss werden die Daten von elektronischen

Datensystemen (IBM SPSS Statistics) erfasst und statistisch ausgewertet. Nach Beendigung der Studie werden alle Daten nach den derzeit gültigen Vorschriften entsprechend gespeichert, archiviert und für eine weitere Verwendung gesperrt.

Deine Daten, erhoben über Fragebogen und Befragungen, werden über einen Zeitraum von 10 Jahren gemäß wissenschaftlichen Standards in einem sicheren System gespeichert und im Anschluss gelöscht, sofern gesetzliche Gründe nicht eine längere Speicherung vorschreiben.

Die Bearbeitung der erhobenen Daten erfolgt in Verantwortung von Prof. Dr. Anja Görtz-Dorten (Ausbildungsinstitut für Kinder- und Jugendlichenpsychotherapie an der Uniklinik Köln (AKiP), Pohligstr. 9, 50969 Köln). Du hast das Recht, Auskunft und Einblick in die zu dir im Rahmen der Studie erfassten personenbezogenen Daten zu nehmen sowie eine unentgeltliche Kopie zu erhalten. Solltest du dabei Fehler feststellen, hast du das Recht, diese korrigieren zu lassen. Du hast außerdem das Recht auf Einschränkung („Sperrung“) der weiteren Datenverarbeitung sowie das Recht auf Datenübertragbarkeit, das heißt das Recht, die dich betreffenden personenbezogenen Daten, die du den Verantwortlichen der Studie bereitgestellt hast, zu erhalten. Damit kannst du beantragen, dass diese Daten entweder dir oder, soweit technisch möglich, einer anderen von dir benannten Stelle übermittelt werden. Zudem hast du das Recht auf Löschung der von dir gespeicherten Daten.

Die pseudonymisierten Daten werden dem *Ausbildungsinstitut für Kinder- und Jugendlichenpsychotherapie an der Uniklinik Köln (AKiP)* und der *Klinik und Poliklinik für Psychiatrie, Psychosomatik und Psychotherapie des Kindes- und Jugendalters der Uniklinik Köln* oder einer von diesen beauftragten Stellen zu wissenschaftlichen Zwecken zur Verfügung gestellt. Im Falle der Veröffentlichung von Studienergebnissen bleibt die Vertraulichkeit deiner persönlichen Daten ebenfalls gewährleistet. Wissenschaftliche Veröffentlichungen von Ergebnissen erfolgen ausschließlich anonymisiert, also in einer Form, die keine Rückschlüsse auf dich zulässt.

Spezifische Information für die Therapie-App

Die Beiträge von dir aus den verschiedenen Anwendungsbereichen der App werden auf dem Smartphone gespeichert und können jederzeit wieder angesehen und gelöscht werden. Die Video-Aufnahmen, die du im Rahmen der App-Nutzung tätigst, können nicht in der gleichen Form wie die übrigen Daten unkenntlich gemacht werden. Die Daten werden mithilfe einer Anleitung von dir selbst auf eine Datenaustauschplattform der Uniklinik Köln namens „Dracoon“ hochgeladen, welche einen sicheren, verschlüsselten und dateibasierten Austausch für sensible Daten ermöglicht. Dafür bekommst du von uns einen individuellen, personalisierten Link zugeschickt. Im Falle eines Leihhandys werden die Daten der Smartphone-App per Kabel auf das IT System der Uniklinik Köln übermittelt. Diese Daten werden jedoch nur von autorisierten Mitarbeitenden der Studie zur wissenschaftlichen Auswertung eingesehen und unter Einhaltung besonderer Schutzmaßnahmen verschlüsselt separat gespeichert. Von den Ergebnissen der Auswertung kann kein Rückschluss mehr auf dich selbst gezogen werden. Die Handydaten werden über einen Zeitraum von 10 Jahren gemäß wissenschaftlichen Standards in einem sicheren System gespeichert und im Anschluss gelöscht, sofern gesetzliche Gründe nicht eine längere Speicherung vorschreiben. Du hast das Recht auf Löschung der von dir erhobenen Handydaten.

9. Sind mit der Datenverarbeitung Risiken verbunden?

Bei jeder Erhebung, Speicherung, Nutzung und Übermittelung von Daten bestehen Vertraulichkeitsrisiken (z.B. die Möglichkeit, die betreffende Person zu identifizieren). Diese Risiken lassen sich nicht völlig ausschließen und steigen, je mehr Daten miteinander verknüpft werden können. Zudem können die Video-Aufnahmen, die du im Rahmen der App-Nutzung tätigst, nicht in der gleichen Form wie die übrigen Daten unkenntlich gemacht werden. Diese Daten werden jedoch nur von autorisierten Mitarbeitenden der Studie zur wissenschaftlichen Auswertung eingesehen und unter Einhaltung besonderer Schutzmaßnahmen verschlüsselt separat gespeichert. Die Auftraggebenden der Studie versichern dir, alles nach dem Stand der Technik Mögliche zum Schutz

deiner Privatsphäre zu tun und Daten nur an Stellen weiterzugeben, die ein geeignetes Datenschutzkonzept vorweisen können. Medizinische Risiken sind mit der Datenverarbeitung nicht verbunden.

10. Kann ich meine Einwilligung widerrufen?

Die Teilnahme an dem Forschungsvorhaben ist ganz und gar freiwillig. Du kannst jederzeit und ohne Angabe von Gründen dein Einverständnis zur Teilnahme zurücknehmen, ohne dass für dich hieraus irgendwelche Nachteile entstehen. In diesen Fällen wird die in unserer Einrichtung bereits laufende Behandlung ohne Studienteilnahme fortgeführt.

Wenn du deine Einwilligung widerrufst, werden keine weiteren Daten mehr erhoben. Die bis zum Widerruf erfolgte Datenverarbeitung bleibt jedoch rechtmäßig.

Im Falle eines Widerrufs werden sämtliche Daten unverzüglich gelöscht oder vollständig anonymisiert.

11. Welche weiteren Rechte habe ich bezogen auf den Datenschutz?

Bei Anliegen zur Datenverarbeitung und zur Einhaltung der datenschutzrechtlichen Anforderungen kannst du dich in erster Linie an die Studienleitung/Auftraggebenden der Studie wenden. Du kannst dich aber auch an folgende Datenschutzbeauftragte wenden:

Datenschutzbeauftragte der Uniklinik Köln: datenschutz@uk-koeln.de, 0221 478 30946

Datenschutzbeauftragte der Universität zu Köln: dsb@verw.uni-koeln.de, 0221-470-0

Du hast außerdem ein Beschwerderecht bei jeder Aufsichtsbehörde für den Datenschutz. Eine Liste der Aufsichtsbehörden in Deutschland findest du unter

https://www.bfdi.bund.de/DE/Infothek/Anschriften_Links/anschriften_links-node.html

Die für die Auftraggebenden der Studie/die Studienleitung zuständige Datenschutzaufsichtsbehörde ist:

Landesbeauftragte für Datenschutz und Informationsfreiheit Nordrhein-Westfalen (Landesbeauftragte für den Datenschutz und Informationsfreiheit, Postfach 20 04 44, 40102 Düsseldorf, Tel.: 0211/38424-0, Mail: poststelle@ldi.nrw.de) wenden.

12. Proband*innen-/Patient*innenversicherung

Da weder studienbedingte Risiken für die Gesundheit bestehen noch studienbedingte Wege anfallen, die über die übliche Regeltherapie hinausgehen, wurden für die Studie keine Proband*innenversicherung und keine Wegeunfallversicherung abgeschlossen. Für die Test-Smartphones wird eine separate Geräteversicherung abgeschlossen.

13. Studienende

Die ambulante Behandlung kann vorzeitig seitens der zuständigen Behandelnden beendet werden, wenn sie nicht notwendig, hilfreich oder ausreichend ist oder wenn krisenhafte Zuspitzungen auftreten, die ggf. auch eine stationäre Behandlung erfordern. Sollten die Einschlusskriterien für die Studie nicht erfüllt sein, aber eine ambulante Behandlung angezeigt sein, kann die Behandlung in unserer Einrichtung ohne Studienteilnahme erfolgen.

14. Aufwandsentschädigung und Kostenerstattung

Es wird keine Aufwandsentschädigung gezahlt. Es entstehen die üblichen Behandlungskosten im Rahmen einer therapeutischen Behandlung. Damit diese von den Krankenkassen übernommen werden, muss ein Antrag an die Kassen gestellt und von dieser bewilligt werden.

15. Information über neue Erkenntnisse

Die Studienmitarbeitenden werden dich rechtzeitig auch über jede Änderung und weitere wichtige, während der Studie bekanntwerdende Information in Kenntnis setzen, die deine Einwilligung zur weiteren Teilnahme beeinflussen könnte.

16. Weitere Fragen?

Solltest du noch weitere Fragen zum Ablauf der Studie, zum Datenschutz, zu deinen Rechten, usw. haben, wende dich bitte an die Studienmitarbeitenden.

Adresse und Telefonnummer des Studienzentrums

Ansprechpartnerinnen: Johanna Schäfer, Lisa Schindler, Alina Huemer & Laura Wähnke
Ausbildungsinstitut für Kinder- und Jugendlichenpsychotherapie
an der Uniklinik Köln (AKiP Köln)
Pohligstr. 9
50969 Köln
E-Mail: jay-forschung@uk-koeln.de
Telefon: 0221 478 87772

Einwilligungserklärung für Jugendliche
(Randomisiertes Kontrollgruppendesign JAY)

- Ich habe die Information gelesen und Ziel, Ablauf und Durchführung der Studie verstanden. Ich wurde mündlich über Wesen, Bedeutung, Tragweite und Risiken der geplanten Studienteilnahme informiert. Mir wurde ausreichend Gelegenheit gegeben, alle offenen Fragen zu klären. Ich habe jederzeit das Recht, weitere Informationen zur Studie zu erfragen.

- Ich erkläre mich freiwillig bereit, an der Studie teilzunehmen.

- Ich bestätige, vollständige und wahrheitsgemäße Angaben zu meiner Krankengeschichte, meines Gesundheitszustandes, zur Einnahme von Arzneimitteln gemacht zu haben sowie weitere Fragen im Zusammenhang mit der Studie wahrheitsgemäß beantwortet zu haben. Ich versichere, jegliche gesundheitlichen Veränderungen den Behandelnden zu melden.

- Ich habe jederzeit das Recht, ohne Angabe von Gründen von der Studie zurückzutreten, ohne dass dadurch Nachteile für meine psychiatrische oder psychotherapeutische Behandlung entstehen.

- Ich wurde darüber informiert, dass alle meine Daten der ärztlichen Schweigepflicht unterliegen.

☐ Ich bin mit dem App-Einsatz inklusive Ton- und Videoaufnahmen einverstanden und erkläre mich dazu bereit, dafür Sorge zu tragen, dass Personen, die der App-Nutzung nicht zugestimmt haben, nicht sichtbar oder hörbar sind.

☐ **Ich habe die Informationen gelesen und verstanden.**

Datenschutz:

Bei dieser wissenschaftlichen Studie werden personenbezogene Daten und medizinische Befunde über Dich erhoben.
Die Speicherung, Weitergabe und Auswertung dieser Daten erfolgt gemäß gesetzlichen Bestimmungen und setzt vor Teilnahme an der Studie die folgende freiwillige Einwilligung voraus:

1. Ich wurde darüber informiert, dass meine Daten in pseudonymisierter Form gespeichert, weitergegeben und analysiert werden.

2. Ich wurde darüber informiert, dass die Daten aus der Videotagebuch-Funktion, welche im Rahmen der JAY Smartphone App erhoben und gespeichert werden, aufgrund der Beschaffenheit der Daten nicht anonymisiert oder pseudonymisiert gespeichert werden können. Der Zugriff auf diese Daten ist jedoch nur autorisierten Projektmitarbeiter*innen der Uniklinik Köln gestattet.

3. Ich erkläre mich damit einverstanden, dass im Rahmen dieser Studie erhobene Daten/Krankheitsdaten auf Fragebogen oder Befragungen in pseudonymisierter Form gespeichert und pseudonymisiert (ohne Namensnennung) weitergegeben werden an die Auftraggebenden der Studie:

Prof. Dr. Anja Görtz-Dorten & Prof. Dr. Manfred Döpfner

Ausbildungsinstitut für Kinder- & Jugendlichenpsychotherapie an der Uniklinik Köln (AKiP)
Pohligstr. 9
50969 Köln

4. Ich erkläre mich damit einverstanden, dass meine Daten nach Beendigung oder Abbruch der Studie bis zu zehn Jahre aufbewahrt werden. Danach werden meine personenbezogenen Daten gelöscht, soweit nicht gesetzliche Aufbewahrungsfristen entgegenstehen.

5. Ich bin darüber aufgeklärt worden, dass ich jederzeit die Teilnahme an der klinischen Studie beenden kann. In diesem Falle werden bereits erhobene Daten gelöscht oder vollständig anonymisiert.

6. Ich habe die Informationen zum Datenschutz zur Kenntnis genommen und willige in die Datenverarbeitung ein.

☐ Ich habe die obigen Informationen zum Datenschutz zur Kenntnis genommen und willige in die Datenverarbeitung ein.

Ich habe die vollständige Teilnehmendeninformation zur Studie sowie ein unterschriebenes Exemplar dieser Einwilligungserklärung erhalten.

____
Vor- und Nachname des/der Jugendlichen (Druckbuchstaben)

__________________________	_________________________
Ort und Datum (persönlich auszufüllen)	Unterschrift des/der Jugendlichen

Ich habe das Aufklärungsgespräch geführt und die Einwilligung der Studienteilnehmenden eingeholt.

____
Vor- und Nachname der Studienmitarbeiterin (in Druckbuchstaben)

__________________________	______________________
Ort und Datum (persönlich auszufüllen)	Unterschrift der Studienmitarbeiterin
